# Adjunctive dexamethasone for the treatment of HIV-infected adults with tuberculous meningitis (ACT HIV): Study protocol for a randomised controlled trial

**DOI:** 10.12688/wellcomeopenres.14006.2

**Published:** 2018-06-20

**Authors:** Joseph Donovan, Nguyen Hoan Phu, Nguyen Thi Hoang Mai, Le Tien Dung, Darma Imran, Erlina Burhan, Lam Hong Bao Ngoc, Nguyen Duc Bang, Do Chau Giang, Dang Thi Minh Ha, Jeremy Day, Le Thi Phuong Thao, Nguyen TT Thuong, Nguyen Nang Vien, Ronald B. Geskus, Marcel Wolbers, Raph L Hamers, Reinout van Crevel, Mugi Nursaya, Kartika Maharani, Tran Tinh Hien, Kevin Baird, Nguyen Huu Lan, Evelyne Kestelyn, Nguyen Van Vinh Chau, Guy E. Thwaites

**Affiliations:** 1 Oxford University Clinical Research Unit, Ho Chi Minh City, Vietnam; 2 Centre for Tropical Medicine and Global Health, Nuffield Department of Medicine, University of Oxford, Oxford, UK; 3 Hospital for Tropical Diseases, Ho Chi Minh City, Vietnam; 4 Pham Ngoc Thach Hospital, Ho Chi Minh City, Vietnam; 5 Cipto Mangunkusumo Hospital, Faculty of Medicine, Universitas Indonesia, Jakarta, Indonesia; 6 Persahabatan Hospital, Faculty of Medicine, Universitas Indonesia, Jakarta, Indonesia; 7 Eijkman Oxford Clinical Research Unit, Eijkman Institute of Molecular Biology, Jakarta, Indonesia; 8 Department of Medicine and Radboud Center for Infectious Diseases (RCI), Radboud University Medical Center, Nijmegen, Netherlands

**Keywords:** Tuberculous meningitis, HIV, Dexamethasone, Drug-induced liver injury, LTA4H, Adrenal suppression, Diabetes, Strongyloides, Hyponatraemia

## Abstract

**Background: **Tuberculous meningitis (TBM) is the most severe form of tuberculosis. Co-infection with HIV increases the risk of developing TBM, complicates treatment, and substantially worsens outcome. Whether corticosteroids confer a survival benefit in HIV-infected patients with TBM remains uncertain. Hepatitis is the most common drug-induced serious adverse event associated with anti-tuberculosis treatment, occurring in 20% of HIV-infected patients. The suggested concentration thresholds for stopping anti-tuberculosis drugs are not evidence-based. This study aims to determine whether dexamethasone is a safe and effective addition to the first 6-8 weeks of anti-tuberculosis treatment of TBM in patients with HIV, and investigate alternative management strategies in a subset of patients who develop drug induced liver injury (DILI) that will enable the safe continuation of rifampicin and isoniazid therapy.

**Methods: **We will perform a parallel group, randomised (1:1), double blind, placebo-controlled multi-centre Phase III trial, comparing the effect of dexamethasone versus placebo on overall survival in HIV-infected patients with TBM, in addition to standard anti-tuberculosis and antiretroviral treatment. The trial will be set in two hospitals in Ho Chi Minh City, Vietnam, and two hospitals in Jakarta, Indonesia. The trial will enrol 520 HIV-infected adults. An ancillary study will perform a randomised comparison of three DILI management strategies with the aim of demonstrating which strategy results in the least interruption in rifampicin and isoniazid treatment. An identical ancillary study will also be performed in the linked randomised controlled trial of dexamethasone in HIV-uninfected adults with TBM stratified by LTA4H genotype (LAST ACT).

**Discussion: **Whether corticosteroids confer a survival benefit in HIV-infected patients remains uncertain, and the current evidence base for using corticosteroids in this context is limited. Interruptions in anti-tuberculosis chemotherapy is a risk factor for death from TBM. Alternative management strategies in DILI may allow the safe continuation of rifampicin and isoniazid therapy.

## Introduction

### Background

*Mycobacterium tuberculosis* causes approximately 10.4 million new cases of tuberculosis and 1.5 million deaths annually, with an additional 0.4 million deaths in individuals co-infected with human immunodeficiency virus (HIV) ^1^. Tuberculous meningitis (TBM) is the most severe form of tuberculosis, killing around 30% of all sufferers despite appropriate anti-tuberculosis chemotherapy ^2^. TBM is especially common in young children and in those with advanced immunodeficiency secondary to HIV, and is characterised by a slowly progressive meningo-encephalitis with necrotising granulomatous inflammation predominantly affecting the basal meninges.

### Treatment of TBM

Rifampicin, isoniazid, pyrazinamide and ethambutol are recommended in current international guidelines for the treatment of drug-susceptible TBM, in adults with or without HIV, with treatment recommended for 9–12 months ^3, 4^. The treatment of drug resistant TBM is more challenging. Adjunctive anti-inflammatory treatment with corticosteroids (dexamethasone) has been shown to improve survival in TBM, in predominantly HIV-uninfected individuals in a small number of trials ^5^.

## Complications of TBM

### Neurological complications of TBM

Hydrocephalus, stroke, and tuberculoma formation are important complications of TBM ^2^. They generally present within the first 3 months of treatment and can be fatal if not detected and treated quickly. Little evidence exists to help guide the management of these complications, which are common in HIV-infected and uninfected TBM patients of all ages ^2^. In untreated HIV-infected patients with TBM, contemporaneous anti-tuberculosis treatment and anti-retroviral therapy (ART) can result in neurological inflammatory complications secondary to ART-driven IRIS ^6^. Tuberculoma and spinal radiculomyelitis are the commonest IRIS complications described.

### Drug induced liver injury (DILI)

Hepatitis is the most common drug-induced serious adverse event associated with anti-tuberculosis treatment, occurring in approximately 10% of HIV-uninfected and 20% of HIV-infected patients ^7^. Almost all episodes occur in the first 3 months of anti-tuberculosis therapy, a critical time in TBM treatment. Rifampicin, isoniazid, and pyrazinamide can all cause DILI, although determining which drug is responsible in individual patients can be difficult. Anti-tuberculosis DILI is widely defined as elevation of blood transaminase concentrations ≥3 times the upper limit of normal (ULN) with symptoms, or ≥5 times the ULN without symptoms ^4^. US Centers for Disease Control and Prevention guidelines for pulmonary tuberculosis suggest stopping all anti-tuberculosis drugs in DILI until transaminase concentrations have returned to <2 times the ULN, followed by sequential re-introduction ^4^, however this approach is probably unsafe in those with TBM. We have previously shown that interruptions in first-line anti-tuberculosis chemotherapy for any reason is an independent risk factor for death from TBM ^8^. The suggested concentration thresholds for stopping anti-tuberculosis drugs are not evidence-based. The optimal strategy for managing DILI in TBM is uncertain ^9^. The majority of asymptomatic rises in transaminases (even those >5 times the ULN) will resolve spontaneously, therefore higher thresholds for stopping therapy, perhaps up to 10 times ULN, may be more appropriate. The optimal order and method of drug re-introduction is unknown, and no randomised comparisons have ever been published within the context of TBM treatment.

### Dexamethasone induced adrenal suppression

Iatrogenic administration of exogenous corticosteroids is associated with adrenal suppression, and the sudden cessation of treatment can lead to Addisonian crises which can be life-threatening. For this reason it is common practice to prescribe corticosteroids in tapering doses, to allow recovery of the adrenal cortex during the treatment period. Thus, by the time the corticosteroid is finished, normal endogenous cortisol production will have resumed with no risk of Addisonian crisis. Whether the use of corticosteroids in the doses prescribed in infectious diseases are associated with significant adrenal suppression is not clear and has not been investigated.

## HIV-associated TBM

### Co-treatment of HIV and tuberculosis

HIV infection increases the risk of an individual infected with *M. tuberculosis* developing TBM, complicates treatment, and substantially worsens outcome ^10– 12^. Co-treatment of HIV and tuberculosis is complex because of the adherence demands of multidrug therapy for two infections, drug-drug interactions between rifampicin and ART, overlapping side effect profiles of anti-tuberculosis drugs and ART, and the frequency of IRIS. The optimal strategy for starting ART in TBM is unclear.

### Use of adjunctive dexamethasone

How corticosteroids confer a survival benefit, and whether they do so in HIV-infected patients with TBM, remains uncertain. In TBM dexamethasone may reduce the early intracerebral inflammatory response, prevent hydrocephalus infarction and tuberculoma formation, and reduce the incidence of neurological IRIS. Dexamethasone may reduce the risk of DILI and thereby improve outcome by enabling uninterrupted anti-tuberculosis treatment. The current evidence-base for using adjunctive corticosteroids for the treatment of HIV-associated TBM is restricted to 98 adults recruited to a trial in Vietnam ^8, 13^. This trial randomised a total of 545 subjects (98 of them HIV-positive) and reported an overall reduction in 9-month mortality due to dexamethasone from 41.3% (112/271) to 31.8% (87/274) (hazard ratio of time to death 0.69; 95% confidence interval (CI) 0.52–0.92, P=0.01). While there was no clear evidence of treatment effect heterogeneity according to HIV status, the number of included HIV-infected subjects was low and the observed benefit in that subgroup was smaller: 61.4% (27/44) in the dexamethasone group died, compared to 68.5% (37/54) in the placebo group (hazard ratio of time to death 0.86; 95% CI 0.52–1.41; P=0.55). On the basis of these data most international guidelines cautiously recommend dexamethasone should be given for HIV-associated TBM, but all acknowledge the paucity of evidence and the need for additional controlled trial data.

## Study hypotheses

Neurological complications are both common and devastating in TBM. Dexamethasone may reduce complications arising from an early intracerebral inflammatory response, including neurological IRIS. Dexamethasone has been shown to improve survival in HIV-unaffected individuals with TBM. **Our primary hypothesis is that adjunctive dexamethasone increases survival from TBM in HIV co-infected adults.** The secondary hypothesis is that current guidelines for the management of anti-tuberculosis DILI in those with TBM result in the premature interruption of rifampicin and isoniazid (the critical active drugs in early therapy) and are thereby placing participants at risk of poor outcomes.

## Study aims

### Primary aim

Our primary aim is to determine whether dexamethasone is a safe and effective addition to the first 6–8 weeks of anti-tuberculosis treatment of TBM in patients with HIV with dexamethasone duration depending on Medical Research Council (MRC) grade ( Supplementary File 1) at the start of treatment. In making this assessment we not only determine whether dexamethasone improves survival, but also whether it lengthens the time to new neurological events, IRIS, drug-related adverse events, opportunistic infections, and disability assessed by the modified Rankin scale ( Table 1). We will follow participants for 24 months to assess longer-term neurological outcomes and the incidence of HIV-associated malignancy in the two treatment arms.

**Table 1.** Modified Rankin scale.

Score	Description
0	No symptoms
1	Minor symptoms not interfering with lifestyle
2	Symptoms that lead to some restriction in lifestyle, but do not interfere with the patients ability to look after themselves
3	Symptoms that restrict lifestyle and prevent totally independent living
4	Symptoms that clearly prevent independent living, although the patient does not need constant care and attention.
5	Totally dependent, requiring constant help day and night.
6	Death

### Secondary aim

Our secondary aim is to investigate alternative management strategies in a subset of patients who develop DILI that will enable the safe continuation of rifampicin and isoniazid therapy whenever possible.

### Design and setting

**Figure 1.**
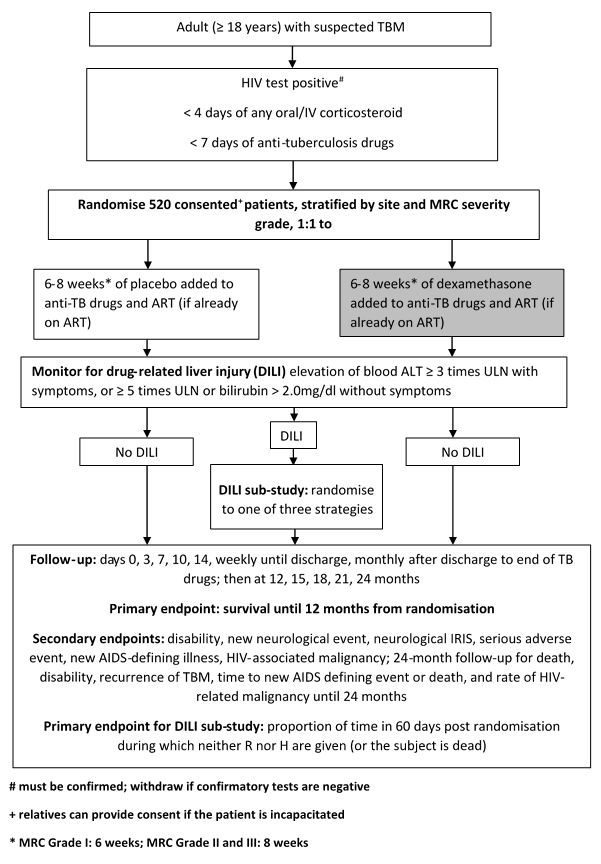
Trial Schema (main trial).

ACT HIV is a parallel group, randomised (1:1), double blind, placebo-controlled multi-centre Phase III trial, comparing dexamethasone versus placebo for 6–8 weeks in addition to standard anti-tuberculosis and antiretroviral treatment. The trial will be set in two hospitals in Ho Chi Minh City, Vietnam and two hospitals in Jakarta, Indonesia. The trial will enrol 520 HIV-infected adults (≥ 18 years old) admitted to participating hospitals with a suspected diagnosis of TBM, as judged by the attending physician, and requiring immediate anti-tuberculosis treatment. Doctors making the diagnosis of TBM will all be senior physicians specialising in either infectious diseases or tuberculosis and lung diseases. All will receive additional diagnostic training and follow a diagnostic standard operating procedure. This trial schema is shown in Figure 1.

An ancillary DILI strategy study will perform a randomised comparison of management strategies in DILI early in anti-tuberculosis treatment (the intensive phase). We will perform an open, randomised comparison of three management strategies with the aim of demonstrating which strategy results in the least interruption in rifampicin and isoniazid treatment. All patients enrolled in the main trial will be eligible to take part in this study, with the exception of those known to have TBM caused by isoniazid resistant or MDR *M. tuberculosis*.

### Ancillary studies

Seven ancillary studies will be conducted within the ACT HIV trial. Some of the studies will only involve a subset of patients recruited at the Hospital for Tropical Diseases (HTD), Ho Chi Minh City, Vietnam. Ancillary studies 2, 3 and 7 will be retrospective data analyses. Ancillary study 4 and 5 will collect and review patient results as the trial progresses. For ancillary study 6 results will be fully analysed at the end of the trial, however laboratory values that may influence clinical care will be available to the clinical team as the trial progresses.

The studies are as follows:

**Ancillary study 1:** A randomised comparison of management strategies in response to DILI (as above). (All patients.)
**Ancillary study 2:** Host and bacterial genetic determinants of treatment response. We hypothesise that the LTA4H gene, and genes involved in related inflammatory pathways, may additionally influence participant inflammatory state, TBM severity, and treatment response. We will also examine genetic variants associated with the development of DILI. (All patients.)
**Ancillary study 3:** Impact of dexamethasone on CSF inflammation and gross cerebral pathology (assessed by serial brain magnetic resonance imaging (MRI)). We will investigate how dexamethasone influences the resolution of inflammatory markers in the CSF and the gross pathological consequences of TBM on the brain (hydrocephalus, stroke, and tuberculoma formation). (HTD patients only.)
**Ancillary study 4:** Influence of diabetes mellitus on presentation and response to treatment. We will investigate whether diabetes mellitus influences clinical presentation and CSF inflammatory phenotype (linking with ancillary study 2), and how it impacts upon treatment outcomes. (All patients.)
**Ancillary study 5:** Influence of Strongyloides infection on presentation and response to treatment. We will determine whether Strongyloides co-infection alters clinical and/or CSF inflammatory phenotype at TBM presentation (linking with ancillary study 2) and treatment response. (All patients.)
**Ancillary study 6:** Pathophysiology and treatment of hyponatraemia and raised intracranial pressure. We will investigate the pathophysiology of TBM-associated hyponatraemia, enabling a better understanding of the causes of hyponatraemia, the relationship between plasma sodium and elevated intracranial pressure, and the best management of severe hyponatraemia. (HTD patients only.)
**Ancillary study 7:** Dexamethasone induced adrenal suppression. We will investigate whether the use of corticosteroids in the doses prescribed in infectious diseases are associated with significant adrenal suppression. (HTD patients only.)

## Endpoints

### Primary endpoint – ACT HIV (main trial)

The primary endpoint of the main trial is overall survival, i.e. the time from randomisation to death, during a follow-up period of 12 months. Survivors known to be alive at 12 months will be censored at that time-point and subjects who withdrew or were lost to follow-up before 12 months will be censored at the date they were last known to be alive.

### Primary endpoint – DILI strategy study

In the DILI strategy study the primary endpoint is the proportion of time in the 60 days following randomisation during which neither rifampicin nor isoniazid are given (or the subject is dead).

### Secondary endpoints – ACT HIV (main trial)

The secondary endpoints of the main trial are as follows:

a) Neurological disability at day 30 from randomisation, monthly until completion of anti-tuberculous drugs, and at months 12, 18 and 24. The main endpoint is the 12-month assessment and subjects who died before 12 months will be treated as having a score of 6 (‘dead’). Neurological disability will be assessed by the modified Rankin score ( Table 1).
b) Time to first new neurological event or death during a follow-up period of 12 months. A neurological event is defined as a fall in Glasgow Coma Score (GCS) by ≥2 points for ≥2 days from the highest previously recorded GCS (including baseline), or the onset of any of the following clinical adverse events: cerebellar symptoms, focal neurological signs, or seizures.
c) Time to neurological IRIS events from randomisation until 6 months. We will follow the International Network for the Study of HIV associated IRIS (INSHI) case definition of IRIS ( 14, Supplementary File 2). Rate of neurological IRIS is defined as the number of IRIS events divided by the observed person-time of follow-up in each treatment group.
d) Time to new acquired immunodeficiency syndrome (AIDS)-defining event (as per World Health Organisation classification) or death, during a follow up period of 12 months.
e) Time to HIV-associated malignancy from randomisation until 12 months. Rate of HIV-associated malignancy is defined as the number of events of the three major HIV-associated malignancies (Kaposi sarcoma, high grade B-cell non-Hodgkin lymphoma and invasive cervical cancer) divided by the observed person-time of follow-up in each treatment group.
f) Serious adverse events until 12 months. Comparison of the frequency of serious adverse events between treatment groups will form an important part of the study analysis.
g) Endpoints assessed at 24 months of follow up. All participants will continue to be followed up for 24 months and the following outcomes will be reported once this period has been completed for all participants: overall survival until 24 months, neurological disability at 24 months, recurrence of TBM until 24 months, time to new AIDS defining event or death until 24 months, and rate of HIV-related malignancy until 24 months.

### Secondary endpoints – DILI strategy study

The secondary endpoints of the DILI strategy study are as follows:

a) Development of acute liver failure (defined as new onset coagulopathy (International normalised ratio (INR)>1.5) and hepatic encephalopathy) after randomisation.
b) ART interrupted due to drug-related injury.
c) Time to new neurological event (defined as a fall in GCS of ≥2 points for ≥48 hours, new focal neurological sign, or new onset of seizures) or death from randomisation until the 12 month follow-up of the main trial.
d) Overall survival, i.e. time to death from any cause, until the 12 month follow-up of the main trial.
e) Neurological disability at the 12 month follow-up of the main trial.

## Inclusion and exclusion criteria

### Inclusion criteria

Study participants for ACT HIV must be adults (aged 18 years or older), HIV-infected, with a clinical diagnosis of TBM (≥5 days of meningitis symptoms, and CSF abnormalities) and anti-tuberculosis chemotherapy either planned or started by the attending physician. Participants will be considered eligible for enrolment in this trial if they fulfil all the inclusion criteria and none of the exclusion criteria. TBM is a serious infection whose standard treatment requires hospitalisation. Therefore all eligible participants will be treated in hospital, at least for the initial 3 weeks of their illness. Pregnant participants are eligible for enrolment. Participants with drug resistant TBM are an important sub group, and are eligible for enrolment into the main study (but not the DILI ancillary study).

Study participants for the DILI strategy study must be receiving first-line anti-tuberculosis drugs and fulfil the definition of drug-related liver injury: elevation of blood transaminase concentrations ≥3 times the ULN with symptoms and signs of hepatitis (vomiting, abdominal pain, jaundice), or ≥5 times the ULN or a rise in serum bilirubin >2.0mg/dL (>34 µmol/L) without symptoms ^4^, and less than 90 days of anti-tuberculosis drugs given.

### Exclusion criteria

Exclusion criteria for ACT HIV are:

a) An additional brain infection (other than TBM) confirmed or suspected: positive CSF Gram or India Ink stain; positive blood or CSF Cryptococcal antigen test; cerebral toxoplasmosis suspected and attending physician wants to give anti-toxoplasmosis treatment with anti-tuberculosis treatment.
b) More than 6 consecutive days of two or more drugs active against *M. tuberculosis* immediately before screening.
c) More than 3 consecutive days of any type of orally or intravenously administered corticosteroid immediately before randomisation.
d) Dexamethasone considered mandatory for any reason by the attending physician.
e) Dexamethasone considered to be contraindicated for any reason by the attending physician.
f) Patient has previously been randomised into this trial for a prior episode of TBM.
g) Lack of consent from the participant or family member (if the participant is incapacitated by the disease).

Exclusion criteria for the DILI strategy study are:

a) TBM known to be caused by isoniazid resistant or MDR *M. tuberculosis* or standard first-line anti-tuberculosis drugs unable to be given for any reason other than DILI.
b) Signs of chronic liver disease of any cause (hepatosplenomegaly, prolonged jaundice, caput medusa, spider angiomata, ascites, oedema).
c) Lack of consent from the participant or family member (if the participant is incapacitated by the disease).
d) Elevation of blood transaminase concentrations ≥3 times the ULN with symptoms and signs of hepatitis (vomiting, abdominal pain, jaundice), or ≥5 times the ULN or a rise in serum bilirubin >2.0mg/dL (>34 µmol/L) without symptoms at baseline (day 0).

## Recruitment, retention and randomisation

### Recruitment and retention

Recruitment activities will only occur in an inpatient hospital setting in participating hospitals. The target sample size of 520 participants will be enrolled into the main trial, with an anticipated accrual rate of 4 years. Once discharged from hospital the participants will be contacted by phone to remind them of their next visit. In addition, patients who have missed a visit will be contacted by phone for a maximum of three times after which a maximum of three home visits can be conducted. All contact attempts will be recorded. As part of routine clinical care participants with suspected TBM will have an HIV test, a lumbar puncture, and a GeneXpert MTB/RIF test on CSF to assess the likelihood of *M. tuberculosis* infection and rifampicin resistance. When possible, participants will be screened for eligibility on the day their CSF results return and at the time the decision is made to start anti-tuberculosis chemotherapy for suspected/confirmed TBM. The name and date of birth of every adult screened for the trial should be added to the site Screening and Randomisation Register, together with the allocated trial number if subsequently randomised, or the reason the participant was not randomised. We anticipate 100 ACT-HIV patients will consent to the ancillary DILI strategy study.

### Randomisation

Randomisation to ACT HIV will be stratified by participating hospital/site and modified MRC TBM severity grade. The randomisation list will be computer-generated based on random permuted blocks with variable block size following Oxford University Clinical Research Unit (OUCRU) standard operating procedures. A 24h web-based randomisation service will be provided. The OUCRU biostatistician in charge of randomisation list preparation will set up statistical code to generate the randomisation list and transfer it to the Study Pharmacist. The Study Pharmacist will then change the random seed, i.e. the initialisation of the random numbers generator, in the statistical code in order to blind the biostatistician and then run the code to prepare the final randomisation list. The generated randomisation lists will be securely incorporated within the trial database. A reliable manual back-up system will also be available. Randomisation to the three strategies for DILI will be 1:1:1 with stratification by initial randomisation (dexamethasone or placebo) and site. Authorised individuals will receive an allocated treatment strategy by visiting the same website used for the primary randomisation.

## Blinding, unblinding and treatment discontinuation

### Blinding

All participants and investigators will be blinded to the treatment allocation. OUCRU clinical trials unit (CTU) pharmacists will create blinded drug packages (in fully made-up and labelled treatment packs) containing either active drugs or identical placebo sufficient for 6–8 weeks of treatment (dependent on the MRC grade of the participant) according to the prespecified randomisation list, and pre-ship them to the sites. After randomisation, the ward or trial research nurse will take the completed prescription form to the site pharmacy; they will dispense the trial-number specific pack containing the study drug. Unused drug will be returned to the site pharmacy if a participant withdraws from treatment. In ancillary study 3 MRI brain images will be read by an independent neuroradiologist, blind to the treatment allocation and outcomes of the participant.

### Unblinding

If, in the opinion of the local clinician, it is important for good clinical care to unblind treatment the documented request will be discussed with the site Principal Investigator (PI) and Chief Investigator (CI). If it is agreed that knowledge of treatment allocation is essential for the best management of the patient, the unblinding code will be provided by the study pharmacist holding the randomisation list at OUCRU CTU upon documented request from the CI. Generalised clinical deterioration is not sufficient for unblinding, given equipoise about the evidence base supporting the use of dexamethasone regardless of clinical severity. All instances of unblinding will be recorded and reported to the Data Monitoring Committee (DMC) and Trial Steering Committee (TSC).

### Protocol treatment discontinuation

An individual participant may stop study drug early for any of the following reasons: Participant no longer believed to have TBM and all anti-tuberculosis treatment stopped; Confirmatory HIV tests are negative and HIV infection excluded; Unacceptable toxicity or adverse event; Intercurrent illness that prevents further treatment; Any change in the participant’s condition that justifies the discontinuation of treatment in the treating physicians opinion and after discussion with the site PI; Inadequate compliance with the protocol treatment in the judgement of the treating physician; Withdrawal of consent for treatment by the participant.

## Trial management

### Interventions

All participants will receive the standard of care anti-TB drugs and ART drugs according to respective national guidelines. In ART-naive patients, ARTs will be started 6–8 weeks after the start of anti-tuberculosis drugs. Study participants for the main trial will be randomised to receive either dexamethasone or placebo (investigational medicinal product (IMP)) as an extra medication for 6–8 weeks, dependent upon the severity of TBM disease ( Table 2). Dexamethasone/placebo will be dispensed at randomisation from the site pharmacy in intravenous and oral (tablet) formulations. Placebo will be identical in appearance to active drug and dosed and dispensed in the same way. The study drug will be given to participants as early as possible in the treatment of TBM but no later than 7 days from the start of anti-tuberculosis treatment.

**Table 2.** Study drug treatment regimen following randomisation.

	MRC Grade I Daily dexamethasone dose/route	MRC Grades II and III Daily dexamethasone dose/route
Week 1	0.3 mg/kg/24 hrs IV	0.4 mg/kg/24 hrs IV
Week 2	0.2 mg/kg/24 hrs IV	0.3 mg/kg/24 hrs IV
Week 3	0.1 mg/kg/24 hrs IV	0.2 mg/kg/24 hrs IV
Week 4	3mg/24 hrs oral	0.1 mg/kg/24 hrs IV
Week 5	2mg/24 hrs oral	4 mg/24 hrs oral
Week 6	1 mg/24 hrs oral	3 mg/24 hrs oral
Week 7	Stop	2 mg/24 hrs oral
Week 8		1 mg/24 hrs oral

**Figure 2.**
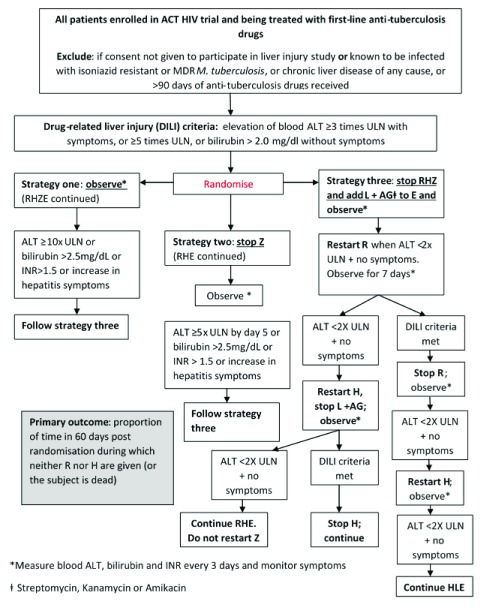
Trial Schema (drug-induced liver injury strategy study).

Participants for the DILI strategy study will be randomised to one of three strategies ( Figure 2): Strategies are as follows:

1) Observe: measure transaminases, bilirubin, and INR every 3 days; do not change/stop anti-tuberculosis drugs unless transaminases rise to ≥10x normal, or total bilirubin rises >2.5mg/dl (>43 µmol/L), or INR >1.5 or symptoms of hepatitis worsen (nausea, vomiting, abdominal pain), in which case go to Strategy 3.
2) Stop pyrazinamide (Z) alone. Observe, measuring transaminases, bilirubin, and INR every 3 days. If transaminases do not fall to <5x ULN by day 5, or total bilirubin rises >2.5mg/dl (>43 µmol/L), or INR >1.5 or symptoms of hepatitis worsen at any time (nausea, vomiting, abdominal pain), go to Strategy 3.
3) Current standard of care (current US CDC guidelines ^4^, Supplementary File 3).

Patients in the DILI study will undergo regular clinical and blood test monitoring, and there is a clear procedure to follow for moving patients from strategy 1 to strategy 3, or from strategy 2 to strategy 3. We hypothesise that current guidelines for the management of anti-tuberculosis DILI result in the premature interruption of rifampicin and isoniazid (the critical active drugs in early therapy) in those with TBM and are thereby placing participants at risk of poor outcomes. Strategies 1 and 2 may demonstrate improved safety compared to strategy 3. However, regular clinical monitoring, and interim review by an independent data monitoring committee, will be in place to ensure safety of participants in case of any increased adverse events in any of the DILI strategy groups.

DILI related interventions are conducted within the ACT HIV trial, but the DILI strategies (specifically arms 1 and 2, compared with the routine care arm 3) are not expected to increase adverse events. It is in fact anticipated that arm 1 (continue all 4 first line anti-TB drugs) and arm 2 (stop only pyrazinamide) of the DILI study will result in less adverse events due to the continuation of the best and most appropriate anti-TB drugs. Liver function tests will be closely monitored. DILI occurs more frequently in individuals with HIV therefore it is very important that DILI interventions are assessed in an HIV positive group, to avoid unnecessary cessation of first line anti-TB drugs if arms 1 and / or 2 are effective. From a safety perspective, if a patient develops DILI, prior randomisation to dexamethasone or placebo should not affect DILI in an adverse way.

### Anti-tuberculosis treatment

First line anti-tuberculosis treatment will follow current Vietnamese and Indonesian national guidelines. Rifampicin (10mg/kg/24 hrs; maximum 600mg), isoniazid (5mg/kg/24hrs; maximum 300mg), pyrazinamide (25mg/kg/24hrs; maximum 2g) and ethambutol (20mg/kg/24 hrs; maximum 1.2g) will be given for at least the first 2 months of treatment, provided drug resistance is not suspected or proven. Pyrazinamide will then be stopped and rifampicin, isoniazid and ethambutol (at the same doses) will then be given until at least 12 months anti-tuberculosis treatment in total has been given. Patients with visual complications will discontinue ethambutol and an alternative drug will be used in its place. The decision of which fourth drug to use will be made by the treating physician, and is not mandated by the trial. In practice this will be either a fluoroquinolone or streptomycin.

For TBM caused by isoniazid-resistant tuberculosis the attending physician should decide which option to take, dependent upon clinical circumstances. For participants with MDR tuberculosis, second-line treatment should be given as soon as possible, following national guidelines and local policies.

Participants with abnormal liver function tests (LFTs) at screening are eligible to enter the main trial. These patients should be given standard first-line anti-tuberculosis treatment unless blood transaminase concentrations are ≥3 times the ULN with symptoms and signs of hepatitis (vomiting, abdominal pain, jaundice), or ≥5 times the ULN or a rise in serum bilirubin >2.0mg/dL (>34µmol/L) without symptoms. In these participants, initial anti-tuberculosis treatment should consist of levofloxacin, ethambutol, and an aminoglycoside (either kanamycin, amikacin or streptomycin). LFTs should be monitored every 3 days and rifampicin started as soon as blood transaminases are <5x the ULN and/or the symptoms and signs of hepatitis resolve. Once the participant is tolerating a rifampicin-containing regimen isoniazid can be introduced and, if isoniazid is tolerated, the aminoglycoside can be stopped. If pyrazinamide is not used in treatment, at least 12 months of anti-tuberculosis treatment must be given. Participants with liver dysfunction at the start of treatment that require modified initial anti-tuberculosis treatment regimens are ineligible for the DILI strategy study.

### Anti-retroviral therapy

ART will be provided for all participants within the current Vietnamese and Indonesian national guidelines. ART in both Ho Chi Minh City and Jakarta is often commenced 6–8 weeks after the start of anti-tuberculosis treatment, with the precise timing left to the discretion of the attending physician. Delays in starting ART of longer than 8 weeks are not encouraged. This practice is supported by our previous trial of immediate versus deferred ART in TBM and consistent with current local practice guidelines ^15^. The optimal time to start ART in TBM is not known, depends upon TBM disease severity and CD4 count, and varies between centres regarding routine practice. Exactly when to start ART is decided upon by the treating physician. A patient with known HIV already on ART will continue this ART when they enter the trial, and during the trial. Whenever possible, patients will be treated with an efavirenz-containing regimen. When viral resistance to first line (NNRTI-based) ART is suspected, case-by-case decisions will be made with regard to HIV-RNA measurement, drug resistance testing, and composition and timing of second line HIV treatment and concurrent tuberculosis treatment, avoiding combined use of rifampicin and protease inhibitors because of strong drug interactions and high risk of toxicity ^16^. Decisions on dose or schedule adjustments for these participants will be made on an individual basis, following the national and local guidelines. If DILI develops ART should not be stopped until all other management has been tried (including interrupting anti-tuberculosis drugs according to the randomised schedule). HIV specialists and liver specialists should be consulted at this point.

### Prophylaxis for opportunistic infections

All participants will receive *Pneumocystis jirovecii* prophylaxis with co-trimoxazole according to national guidelines (CD4 count <200 cells/mm ^3^). In the DILI strategy study prophylactic co-trimoxazole and/or fluconazole should be stopped before all other drugs if DILI develops.

### Use of concomitant medication

All other concomitant medications essential for participant management are permitted at enrolment, subject to the exclusion criteria of no contraindications to the use of dexamethasone in the judgement of the attending clinician. If use of a concomitant medication that cannot safely be used with dexamethasone becomes essential after randomisation, then the IMP should be stopped and the concomitant medication used without unblinding. Drugs which increase the risk of gastrointestinal bleeding, such as non-steroidal anti-inflammatory drugs (NSAIDS), should be used with caution. Any other oral or intravenously administered corticosteroids are not permitted, unless deemed essential, in which case the study drug should be stopped and replaced by the chosen corticosteroid. The treatment of tuberculosis and HIV once the study drug has been stopped should be determined by national guidelines and the physician responsible for the care of the participant.

### Management of neurological complications occurring after the start of anti-tuberculosis treatment

Neurological deterioration, manifest by falling conscious level or new focal neurological signs, is common after the start of treatment of TBM ^2^. Common causes are hydrocephalus, stroke, tuberculomas, and hyponatraemia, and neurological events occurring within 2 months of starting ART may also be due to IRIS. Whenever possible, all patients with unexplained neurological deterioration should have either brain imaging with computed tomography (CT) or MRI. Corticosteroids are not routinely recommended for the treatment of hydrocephalus or stroke. Tuberculomas may cause significant peri-lesional inflammation and oedema and can manifest as new onset seizures, focal neurological deficit, or globally reduced consciousness. In these circumstances, most physicians recommend using corticosteroids. Therefore, if after clinical and neuroradiological review the attending physician believes a patient’s neurological deterioration is due to tuberculoma(s), open-label dexamethasone is recommended. Study drug should be stopped, if it still being given (without the need for unblinding), and high-dose intravenous dexamethasone (0.4mg/kg/24hrs) should be prescribed. The speed of dexamethasone reduction and the total duration of therapy should be determined on a case-by-case basis by the attending physician.

### Management of IRIS

Untreated HIV-infected patients starting ART treatment for the first time after at least 2 weeks of anti-tuberculosis treatment will be at risk of developing neurological IRIS. A single randomised controlled trial demonstrated that corticosteroids reduced the need for hospitalisation and therapeutic procedures and hastened improvements in symptoms, performance, and quality of life in IRIS associated with non-neurological tuberculosis IRIS ^17^. Therefore, in the absence of other data, if the criteria for IRIS are met, then we recommend the participant should be given open-label dexamethasone or prednisolone. If the participant has IRIS with neurological involvement then intravenous dexamethasone 0.4mg/kg/24 hrs should be given. Oral prednisolone (following the published trial regimen ^17^) should be given for non-neurological IRIS (prednisone 1.5mg/kg per day for 2 weeks, then 0.75mg/kg per day for 2 weeks). IRIS will be diagnosed if the participant meets the antecedent requirements, clinical IRIS criteria, and alternative explanations for the clinical deterioration are excluded if possible ( Supplementary File 2). Cases where alternative diagnoses cannot be fully excluded because of limited diagnostic capacity should be regarded as “probable paradoxical tuberculosis-associated IRIS”. In these probable cases, should resolution of clinical or radiological findings of the suspected IRIS episode occur without a change in tuberculosis treatment or ART having been made, they could then be reclassified as ”paradoxical tuberculosis-associated IRIS” cases.

## Data collection

The trial assessment schedule for ACT HIV is outlined in Table 3.

**Table 3.** ACT HIV trial assessment schedule.

	DAYS MONTHS										
	SCREENING	0	3	7	W eekly until discharge	30	60	M onthly to anti-TB drug end	12	18	24
ALL PARTICIPANTS											
Eligibility assessment	(X)										
Participant information sheet and consent	X										
Randomisation		X									
Clinical assessment	(X)	X	X	X	X	X	X	X	X	X	X
Disability assessment						X	X	X	X	X	X
Chest X-ray	(X)						X		X		
Lumbar puncture (with paired plasma glucose) CSF Cryptococcal Ag test	(X) (X)					X	X				
HIV test	(X)										
EDTA blood for genetic tests Full blood count CD4 count HIV viral load Storage for later DNA extraction	(X) (X)	X X X		(X)			X				
Sodium Urea/Creatinine ALT/ bilirubin Hepatitis C antibodies Hepatitis B surface antigen Fasting blood sugar/ HbA1c C-peptide/Lipids Strongyloides serology Serum Storage	(X) (X) (X)	X X X X X X		(X)	(X) (X) (X)		X	(X) (X)			
Stool for Ova, cysts and parasites (Strongyloides) microscopy		X					X				
SUBSET OF PARTICIPANTS RECRUITED TO IMAGING STUDY (HTD Vietnam only)											
Brain MRI		X					X		X		
SUBSET OF PARTICIPANTS RECRUITED TO HYPONATRAEMIA/ICP ANCILLARY STUDY (HTD Vietnam only)											
24-hour fluid balance	(X)	X	X	X	X						
Plasma sodium Plasma osmolality		X	X	X	X						
Urinary sodium Urinary osmolality		X	X	X	X						
Plasma cortisol		X									
Ultra-sound assessment of intravascular volume		X	X	X	X						
Ultra-sound measurement of optic nerve sheath diameter		X	X	X	X						
SUBSET OF PARTICIPANTS RECRUITED TO ADRENAL SUPPRESSION ANCILLARY STUDY (HTD Vietnam only)											
Synacthen test					X (day 21)		X				

### Clinical assessment

Clinical assessment will include conscious level by GCS, new or ongoing focal neurological deficit, clinical treatment response, all serious adverse events including new malignancies and opportunistic infections, all adverse events of any grade leading to modification of anti-tuberculosis treatment or ART or their interruption/early discontinuation (and clinician-assessed likelihood of relationship of adverse event to dexamethasone), and adherence to drugs (study drug, anti-tuberculosis drugs and ART). Assessment of disability by the modified Rankin score will be performed at day 30 from randomisation, monthly until completion of anti-TB drugs, and at months 12, 18 and 24.

### Inpatient assessment

The clinical team will record daily GCS and new focal neurology throughout the participant’s hospital admission. Participants will be visited by one of the research team at screening, baseline, and at least every 3 days for the first 4 weeks of treatment (unless they are discharged or die before 4 weeks) and then at least every 7 days whilst they remain in hospital. Formal trial clinical assessments will occur on day 0, 3, 7, 10, 14, and weekly thereafter until discharge (+/- 1 day).

### Outpatient assessment

After discharge clinical assessments will occur monthly until 12 months. Some of these assessments can be made by phone. Formal outpatient review will occur monthly (+/- 7 days) for at least the first 2 months following hospital discharge. The patient should have formal outpatient review at least every 2 months until month 12 after randomisation. Thereafter patients should be followed up by phone call at 15 months and 21 months and by formal outpatient review at 18 months and 24 months from randomisation.

### HIV monitoring

HIV infection must be confirmed before entry to the study. Peripheral blood CD4 count and HIV viral load will be measured at baseline (routine counts taken within 2 weeks of study entry are acceptable), and CD4 count will be repeated at 60 days.

### Liver function

Alanine transaminase (ALT) and bilirubin will be measured to evaluate liver toxicity every 7 days until discharge.

### Additional blood tests

EDTA blood will be taken for hepatitis B surface antigen and hepatitis C antibodies, and serum will be stored for later DNA extraction where consent has been given. We will use the DNA to investigate novel genetic determinants of treatment response, including LTA4H genotype, and the development of DILI.

### Glycosylated haemoglobin (HbA1c) and fasting blood sugar

HbA1c and fasting blood sugar will be measured at baseline and at 60 days from randomisation. This will enable determination of the frequency of undiagnosed diabetes in those presenting with TBM, assess diabetic control in those known to have diabetes, and evaluate the influence of dexamethasone and anti-tuberculosis treatment on diabetic control over the first 60 days of TBM treatment. To enable more detailed phenotyping of diabetes we will also measure C-peptide and blood lipids at baseline and store plasma for future diabetes related auto-antibody testing.

### Strongyloides

All enrolled participants will be tested for serological evidence of Strongyloides infection (past or latent infection) with stool examination for evidence of active infection at baseline and at 60 days from randomisation. When infection is detected, treatment with ivermectin will be provided. High-dose corticosteroids can lead to re-activation of latent Strongyloides and hyperinfection syndrome. Stool will be examined for Strongyloides larvae on day 60 (end of study drug) to determine whether reactivation alters TBM treatment responses.

### Synacthen test

We will compare adrenal responsiveness at 3 weeks after randomisation and at the end of study drug treatment (6 or 8 weeks depending upon disease severity) in 100 consecutive patients using the short Synacthen test. The patient’s background cortisol level is measured by drawing 2mls of blood at 0900hrs. 250mcg of Synacthen is then administered intravenously; 3ml samples of blood are taken at thirty minutes and sixty minutes to measure the cortisol level after this adrenal stimulation. Synacthen test will be repeated at day 60 after randomisation.

### Lumbar puncture

Lumbar puncture should be performed, unless clinically contraindicated, as part of routine clinical care for the baseline assessment and on days 30 and 60 after randomisation to assess treatment response. Day 60 lumbar punctures are not routine practice in the Jakarta hospitals and will therefore not be mandated by the trial in these centres. Opening pressure should be measured, at least 5mls of CSF should be taken for mycobacterial investigations alone, and assessments of cell count and differential, protein, glucose (paired with serum), and lactate should also be performed on 1–2 mls of additional CSF. Ziehl Neelsen (ZN) stain, GeneXpert, and *M. tuberculosis* culture will be performed on all CSF taken. As part of an ancillary study of the impact of dexamethasone on CSF inflammation and gross cerebral pathology we will measure concentrations of a variety of inflammatory mediators in the CSF (leucocytes, cytokines, chemokines, and eicosanoids, for example) at baseline and on days 30 and 60 after randomisation to determine how dexamethasone influences their expression.

### Hyponatraemia and raised intracranial pressure

In the subset of participants enrolled to HTD, Ho Chi Minh City, Vietnam, we will investigate the pathophysiology of TBM-associated hyponatraemia by serial assessments of fluid balance, paired plasma and urinary sodium and osmolality, and intravascular volume by Doppler ultrasound assessment of inferior vena cava collapsibility index ^18, 19^. We will also use portable ultrasound to measure the optic nerve sheath diameter, which has been shown to be a reliable and non-invasive measure of raised intracranial pressure ^20, 21^. These additional measurements will be assessed at days 0, 3, and 7, and weekly until discharge. Plasma cortisol will be measured at day 0.

### Imaging

Chest Xray will be performed at screening and on day 60 and 12 months after randomisation. In the subset of participants enrolled to HTD, Ho Chi Minh City, Vietnam, we will perform brain imaging by MRI (or CT if the participant cannot tolerate an MRI) at baseline (+/- 7 days) and at 60 days (+/- 7 days) and at 12 months (-0/+1 month). At sites other than HTD, Ho Chi Minh City, Vietnam, brain imaging is performed based upon the routine care at that site. Brain imaging may not always be readily available, and this study does not mandate brain imaging for every patient. Brain imaging for suspected TBM is strongly supported, and individual decisions will be made by the treating physician.

## Adverse events and safety reporting

### Adverse events

Specific procedures will be followed when notifying and reporting adverse events (AEs) or adverse reactions (ARs). The definitions of the EU Directive 2001/20/EC Article 2 based on the principles of ICH good clinical practice (GCP) apply to this trial protocol. All AEs and ARs will be assessed as to whether they are serious or not. If the event is serious and not only related to TBM, or is fatal, then a serious adverse event (SAE) form must be completed and the OUCRU CTU notified within 24 hours. All AEs and ARs (serious and non-serious) should be graded using toxicity gradings.

Causality of all SAEs or serious adverse reactions (SARs) in relation to the trial therapy (dexamethasone) will be assessed. There are five categories: unrelated, unlikely, possible, probable, and definitely, related. If the causality assessment is unrelated or unlikely to be related, the event is classified as an SAE. If the causality is assessed as possible, probable or definitely related, then the event is classified as an SAR. If there is at least a possible involvement of the trial treatment (or comparator), the investigator must assess the expectedness of the event. An unexpected adverse reaction is one not previously reported in the current Summary of Product Characteristics (SPC) at the time the event occurred, or one that is more frequent or more severe than previously reported. If a SAR is assessed as being unexpected, it becomes a suspected unexpected serious adverse reaction (SUSAR). Investigators should always check the current version of the SPC.

### Safety reporting

The OUCRU CTU is responsible for the reporting of SUSARs and other SARs to the regulatory authorities and the research ethics committees. The following events will be reported to the relevant authorities in Vietnam and Indonesia: All unexpected SAEs, all SAEs judged to be related or possibly related to the trial intervention, and all deaths. All SAEs will be reported to OxTREC (Oxford Tropical Research Ethics Committee) in the annual review form and to the DMC in accordance to the DMC charter. An independent DMC will oversee the safety of the trial.

### Interim analyses

Interim analyses are planned after 6 and 12 months of recruitment and yearly thereafter until the completion of the trial but the DMC has the authority to modify the frequency of interim analyses. At these interim analyses, the DMC will receive a report including unblinded summaries of baseline characteristics, the primary endpoint, and adverse events by treatment arm. The DMC will also review data from those enrolled into the DILI strategy ancillary study, in particular the incidence of acute hepatic failure in each of the management strategy arms.

## Statistical analysis

### Sample size justification – ACT HIV (main trial)

A previous trial of dexamethasone included 545 subjects with TBM and reported a reduced risk of death by 9 months associated with dexamethasone (hazard ratio of death 0.69 (95% CI 0.52–0.92)) and no evidence of heterogeneity with respect to HIV status though the number of HIV-infected patients was low (98 subjects) and none of them received antiretroviral therapy ^8^. A recent TBM trial of intensified TBM treatment reported 9-month mortality in 349 HIV-infected patients of 40% in both study arms and all subjects received dexamethasone ^7^. Few additional deaths are expected to occur between months 9 and 12 ^15^. Assuming a target hazard ratio of 0.69 (corresponding to a mortality reduction from 52% to 40% in favour of dexamethasone), a total of 229 deaths need to be observed during the 12-month follow-up duration to obtain a power of 80% at the two-sided 5% significance level according to Schoenfeld’s formula. To achieve this and allowing for a 5% loss-to-follow-up, a total of 520 HIV-infected subjects with TBM need to be recruited into the main trial.

### Sample size justification – DILI strategy study

A review of 36 subjects who interrupted rifampicin and isoniazid because of clinical hepatitis or jaundice events from our previous trial ^7^ gave the following data: the median (IQR) onset date of the DILI was 50 (15–84) days from initiation of anti-tuberculosis treatment, the median (IQR) duration of the rifampicin and isoniazid interruption was 16 (12–24) days, and 12 subjects subsequently died (8 of them within 60 days). Of note, the duration of the treatment interruption was <30 days for 32 (89%) of the 36 subjects and the remaining 4 subjects never re-started rifampicin or isoniazid but continued to receive alternative anti-tuberculosis treatment for >100 days. For the DILI strategy study we hypothesise that strategies 1 and 2 will result in a relative reduction in the duration of the treatment interruption of 50% for subjects with interruptions <30 days, but that they do not affect longer interruptions (as the corresponding subject might have permanent intolerance to rifampicin and isoniazid) or mortality. Based on simulations of hypothetical trials using re-sampling from the data described above, the hypothesised treatment effect, and the Wilcoxon rank sum test for analysis, we determined that the power to detect such an effect size with a sample size of at least 50 subjects per arm is >85%. Of note, given this is an ancillary and essentially ‘opportunistic’ study, we have chosen a liberal (i.e. not multiplicity corrected) two-sided significance level of 5% for each of the two primary comparisons of strategies 1 and 2, respectively, versus strategy 3. A total of 170 participants will be recruited to the DILI sub-study. The DILI sub-study has been powered for the combined analysis of ACT HIV (we anticipate 100 ACT HIV patients will consent to the DILI sub-study) and LAST ACT (a linked RCT of dexamethasone in HIV-uninfected adults with TBM stratified by LTA4H genotype, from which we anticipate 70 patients will enter the DILI sub-study) ^22^.

### Analysis populations – ACT HIV (main trial)

Patients in the main trial will be analysed according to their randomised arm as an intention-to-treat (ITT) analysis. In addition, the primary endpoint will be analysed in the per-protocol population, which will exclude the following patients: patients with a final diagnosis other than TBM, major protocol violations and those receiving less than 1 week of administration of the randomised study drug for reasons other than death. Published diagnostic criteria ^23^ will be applied to all enrolled participants at the end of the study when all mycobacterial culture results are available ( Supplementary File 4). The criteria will sub-divide all cases into definite, probable and possible TBM, and those with an alternative diagnosis.

For the primary analyses of the main trial the second randomisation in the DILI strategy study will be ignored and the estimated dexamethasone treatment effect can thus be interpreted as an average effect across these three management strategies. We believe that this is justified because only approximately 100 (19%) subject are expected to be enrolled in the nested trial with roughly similar numbers from both arms, because the efficacy of the different management strategies is unlikely to depend on whether the patient received dexamethasone or not as it tests a very different intervention, and because the anticipated effect of the management strategy on survival is relatively small. However, in a supplementary analysis, we will also compare the primary endpoint between the treatment policies “dexamethasone treatment plus standard of care management of drug-related liver injury” vs. “placebo treatment plus standard of care management of drug-related liver injury” using an inverse probability weighting based analytical framework ^24^.

### Analysis populations – DILI strategy study

All patients in the DILI strategy study will be analysed according to their randomised arm as an ITT analysis. The two primary comparisons are the comparisons of strategies 1 and 2, respectively, versus strategy 3 (with tests conducted at the unadjusted two-sided 5% significance level) and comparisons between strategies 1 and 2 will be exploratory only.

### Primary endpoint analysis – ACT HIV (main trial)

The primary endpoint of the main trial is overall survival, i.e. time from randomisation to death, during 12 months of follow-up. Overall survival will be analysed with a Cox proportional hazards regression model with treatment as the only co-variate and stratification by TBM MRC severity grade at enrolment (I, II, or III) and country (Vietnam or Indonesia). The primary effect measure is the resulting hazard ratio comparing dexamethasone vs. placebo with a corresponding two-sided 95% confidence interval and p-value. The significance level of the associated two-sided test will be set to 5%. Kaplan-Meier plots and explicit survival estimates at 3, 6, 9, and 12 months of follow-up will also be calculated for the full populations and in the subgroups defined by TBM disease severity and country separately. The proportional hazards assumption will be formally tested based on scaled Schoenfeld residuals and visually assessed by a plot of the scaled Schoenfeld residuals versus transformed time. In case of a significant test, a formal comparison of the absolute risk of death at 12 months between the two groups will also be performed (using a Wald-type test based on Kaplan-Meier estimates at 12 months and associated standard errors using Greenwood’s formula).

The homogeneity of the treatment effect on overall survival across subgroups will be assessed by subgroup analyses and formal tests of interaction between treatment and the following grouping variables: TBM MRC severity grade at enrolment (I, II, or III), country (Vietnam or Indonesia), drug resistance pattern (MDR-TB or rifampicin mono-resistance, isoniazid resistant non-MDR, no or other resistance), ART status at enrolment (ART naive, ≤3 months of ART, >3 months of ART), and CD4 cell count at enrolment. To obtain an adjusted treatment effect estimate and to assess the effect of other covariates on survival, the primary endpoint will also be modelled using a multivariable Cox proportional hazards regression model including the following covariates (in addition to the treatment group): TBM MRC severity grade at enrolment, country, drug resistance pattern, ART status and CD4 cell count at enrolment. Multiple imputation will be used to handle missing covariates.

### Primary endpoint analysis – DILI strategy study

For the analysis of the primary endpoint of the DILI strategy study, the non-parametric Wilcoxon rank sum test will be used for pairwise comparisons. An additional adjusted analysis (with adjustment for the initial randomisation, HIV-status, and the time from initial randomisation to the second randomisation) will be also be performed treating the outcome as an ordinal outcome and using a proportional odds logistic regression model (which can be interpreted as an extension of the Wilcoxon rank sum test). 

### Secondary endpoint analysis

For ACT HIV, neurological disability (assessed by modified Rankin scale) at 12 months will be compared between the two arms with a proportional odds logistic regression model with the treatment assignment as the main covariate and adjustment for TBM MRC severity grade, and country. The result will be summarised as a cumulative odds ratio with corresponding 95% CI and p‐value. Patients with a missing 12-month disability assessment will be excluded from the main analysis but an alternative analysis based on multiple imputation (including disability assessments at earlier time points in the imputation model) will also be performed. Secondary time-to-event endpoints (time to neurological event or death, time to new AIDS event or death) will be analysed in the same way as the primary endpoint. The number of IRIS and HIV-associated malignancy events in each group will be summarised and the event rate calculated in each arm. Comparisons of the rates between the treatment arms will be based on a cause-specific proportional hazards model of the time to the first IRIS event (or HIV-associated malignancy, respectively) or death with treatment as the only covariate.

### Analysis of adverse events

The number of patients with any adverse events and specific events, respectively, will be summarised and informally compared between the two treatment arms based on Fisher’s exact test. The total number of adverse event episodes per patient will also be summarised and informally compared based on a quasi-Poisson regression model with treatment as the only covariate. The following subgroups of adverse events will also be separately summarised: grade 3&4 adverse events; serious adverse events; serious adverse events possibly, probably, or definitely related to the study drug; adverse events leading to TB treatment or ART interruptions. Grade 3&4 laboratory abnormalities will be summarised in the same way as clinical adverse events.

Adverse events due to DILI will be recorded for the DILI study but also for the primary intervention (dexamethasone or placebo). An increased number of adverse events in DILI arms 1 and 2 are not expected, but if they do occur these adverse events will be associated with both the DILI randomisation strategy and for the primary intervention arm in which they occurred.

#### Analysis of ancillary studies

Ancillary studies will be analysed within the same groups as allocated based on the dexamethasone intervention. Allocation to the intervention (dexamethasone or placebo) is important for each sub study, given the effect of corticosteroids on inflammatory pathways, diabetes control, Strongyloides infection, raised intracranial pressure and adrenal suppression. DILI study patients will be analysed in their DILI arm, but an additional adjusted analysis (with adjustment for the initial randomisation) will also be performed.

### Baseline descriptive analyses

Baseline characteristics will be summarised as median (lower and upper quartiles) for continuous data and frequency (percentage) for categorical data. The amount of missing data for each baseline characteristic will also be displayed. 

## Ethical considerations

### Treatment

All participants will receive the best available treatment of tuberculosis and HIV, following local and national guidelines. Corticosteroids are commonly prescribed drugs and there is widespread experience and expertise concerning their safe use. The choice (dexamethasone), dose, route of administration and duration of study treatment follows the international guidance for the treatment of HIV-uninfected participants with TBM. Previous trials have demonstrated the safety of this or similar regimens. In particular, adjunctive corticosteroids were not associated with increased gastro-intestinal bleeding in a meta-analysis of all published TBM trials, although participants in this trial will be carefully monitored for this event. There is a possibility dexamethasone may increase the risk of HIV-associated malignancies, which will also be monitored closely in the trial.

### Confidentiality

Participants’ confidentiality will be maintained throughout the trial. Participants will be assigned a trial identification number and this will be used on case report forms (CRFs); participants will not be identified by their name. The investigator will keep securely a participant trial register showing identification numbers, surnames and date of birth. The unique trial number will identify all laboratory specimens, case record forms, and other records and no names will be used, in order to maintain confidentiality. Data submitted to OUCRU CTU and samples sent to central testing facilities will be identified only by the trial number and participant initials.

### Consent

Written informed consent must be obtained in order to enter into the trial and be randomised. If a participant lacks capacity, written consent must be obtained from a person with responsibility (e.g. family member/relative), in their own language before enrolment by the site PI or an appropriately trained doctor. All potential participants (or their families) will be given a participant information sheet clearly listing the risks and benefits of the trial. All potential participants (or their families) will be able to discuss participation with their consulting doctor who will be able to address questions not covered or arising from the participant information sheet. Incapacitated adults with TBM will be eligible to enter the trial. These adults have more severe disease and therefore may benefit most from adjunctive dexamethasone. We anticipate around 70% of participants with TBM will lack capacity at the start of treatment. An option will be given to patients to enrol in the main study, but not the DILI strategy study.

If consent is provided by a relative, the participant should be consulted and consent recorded if and when they have the capacity to do so. If they are happy to remain in the trial, the participant should complete a participant consent form at this time. If they wish to withdraw from the trial, no further trial-related procedures will be performed, but data to this point would be used in analysis. Data from any participant who dies before regaining capacity (but whose family member has provided consent) will be included in analysis.

### Protocol violations

All deviations from protocol will be addressed in source documents and reported to the OUCRU CTU.

### Withdrawing from the trial

A participant (or their relative) is free to refuse to participate in or withdraw from all or any aspect of the trial, at any time and for any reason. If a participant chooses to discontinue their trial treatment they should always be followed up (providing they are willing) and they should be encouraged not to leave the whole trial. If they do not wish to remain on trial follow-up however, their decision must be respected and the participant will be withdrawn from the trial. Participants may change their minds about stopping trial follow-up at any time and re-consent to participation in the trial.

### Data collection and storage

Clinical data and clinical laboratory data will be entered into CliRes, a 21 CFR Part 11-compliant data capture system provided by the OUCRU IT department. The data system includes password protection and internal quality checks, such as automatic range checks, to identify data that appear inconsistent, incomplete, or inaccurate. Trial data will be recorded onto paper CRFs and entered into CliRes. The participants will be identified by a unique trial specific number and/or code in any database. The name and any other identifying detail will not be included in any trial data electronic file. CRFs, clinical notes and administrative documentation will be kept in a secure location and held for 15 years after the end of the trial. Clinical information will not be released without written permission, except as necessary for monitoring, auditing and inspection purposes. Electronic data will be kept for at least 20 years at the OUCRU CTU.

### SPIRIT checklist

A SPIRIT checklist for this trial protocol is attached ( Supplementary File 5).

### Trial Committees

A Trial Management Group (TMG) will be formed to conduct the day-to-day management of the trial at the OUCRU CTU. This will include the CI, Head of OUCRU CTU, Trial Statistician, Clinical Project Manager, Trial Manager, Data Manager and Jakarta trial coordinator. The group will meet at least once per month, although may meet more or less often as required. The TSC has membership from the TMG plus independent members (Professor Nicholas Paton (Infectious Diseases Physician and Clinical Trialist, National University of Singapore, Singapore), Professor Ben Marais (Senior Tuberculosis Researcher and Trialist, University of Sydney, Australia), Dr Truong Huu Khanh (Infectious Diseases Physician, Paediatric Hospital Number 1, Ho Chi Minh City, Vietnam)), including the Chair (Professor Robert Wilkinson (Honorary Professor and Director Wellcome Centre for Infectious Diseases Research in Africa, University of Cape Town, South Africa)). The role of the TSC is to provide overall supervision for the trial and provide advice through its independent chair. The ultimate decision for the continuation of the trial lies with the TSC. The DMC (Professor Sarah Walker (DMC Chair, Senior Statistician and Clinical Trialist, MRC Clinical Trials Unit, University College London), Professor Graeme Meintjes (Senior Infectious Diseases/HIV Physician, University of Cape Town, South Africa) and Professor Nina Ruslami (Senior TBM Clinician and Researcher, Universitas Padjadjaran, Bandung, Indonesia)), will advise the TSC and can recommend premature closure or reporting of the trial, or that recruitment be discontinued or modified. The DMC is independent from the sponsor. Access to interim data and results will be confidential and strictly limited to the DMC and results (except for the recommendation) will not be communicated to the outside and/or clinical investigators involved in the trial. This trial is sponsored by The University of Oxford (Contact: University of Oxford, Research Services, University Offices, Wellington Square, Oxford OX1 2JD, Tel +44 (0) 1865 282585).

## Data dissemination

Manuscripts arising from the trial will, wherever possible, be submitted to peer-reviewed journals which enable Open Access via UK PubMed Central (PMC) within six months of the official date of final publication. In line with research transparency and greater access to data from trials OUCRU’s clinical trials are registered at ClinicalTrials.gov and a data sharing policy is in place. Data exchange complies with Information Governance and Data Security Policies in all of the relevant countries.

## Discussion

TBM remains the most severe form of tuberculosis, and is especially common in those infected with HIV. Whether corticosteroids confer a survival benefit in HIV-infected patients remains uncertain, with the current evidence base for using corticosteroids in this context restricted to 98 adults recruited to a trial in Vietnam ^8, 13^. The ACT HIV trial aims to determine whether dexamethasone is a safe and effective addition to the first 6–8 weeks of anti-tuberculosis treatment of TBM. ACT HIV will be performed in parallel with a randomised double blind placebo controlled trial of adjunctive dexamethasone in HIV-uninfected Vietnamese adults stratified by Leukotriene A4 hydrolase (LTA4H) genotype (LAST ACT, clinical trial registration NCT03100786) ^22^. Both trials will recruit to seven ancillary studies, including a study to investigate alternative management strategies in a subset of patients who develop DILI, enabling safe continuation of rifampicin and isoniazid therapy whenever possible. In recruiting the target 520 patients into the main trial, this trial has the opportunity to also study the following: Host and bacterial genetic determinants of treatment response; Impact of dexamethasone on CSF inflammation and gross cerebral pathology; Influence of diabetes mellitus on presentation and response to treatment; Influence of Strongyloides infection on presentation and response to treatment. Pathophysiology and treatment of hyponatraemia and raised intracranial pressure; Dexamethasone induced adrenal suppression. These data will be valuable in guiding the management of adjunctive corticosteroid therapy in HIV-infected individuals.

## Trial status

Trial protocol version 1.4, 1 ^st^ August 2017. Estimated recruitment start date 1 ^st^ June 2017. Estimated time for recruitment is 4 years.

## Ethical statement

The trial has ethics approval from the Oxford Tropical Research Ethics Committee (approval number 36-16), the Ethics Committees of the Hospital for Tropical Diseases (approval number CS/ND/16/37) and Pham Ngoc Thach Hospital (approval number CS/PT/16/07), the Vietnam Ministry of Health, and Faculty of Medicine University of Indonesia (17-01-0066). Protocol version 1.4 has ethics approval from the Oxford Tropical Research Ethics Committee, the Ethics Committee of the Hospital for Tropical Diseases, the Ethics Committee of Pham Ngoc Thach Hospital, the Vietnam Ministry of Health, and the Faculty of Medicine University of Indonesia.

Informed consent will be obtained for all study participants.

## Data availability

No data are associated with this article.

## Disclosures

Due to the linked nature of this trial, some sections of this protocol also form part of the linked RCT LAST ACT (Trial registration number: NCT03100786), which has also been submitted to *Wellcome Open Research* ^22^. LAST ACT is a parallel group, randomised (1:1), double blind, placebo-controlled, multi-centre Phase III non-inferiority trial, comparing dexamethasone versus placebo for 6–8 weeks in addition to standard anti-tuberculosis treatment in HIV-uninfected patients with TBM stratified by LTA4H genotype. The 7 ancillary studies in ACT HIV are also recruited to through the LAST ACT trial. As such the hypotheses, design, methods, sample size justification; analysis plans and endpoints of these ancillary studies will also be described in the LAST ACT trial, and will appear identically here. ACT HIV follows the same OUCRU protocols and local / national guidelines as LAST ACT, therefore randomisation, blinding and unblinding procedures, adverse event and safety reporting, ethics and confidentiality sections also appear identically here for ACT HIV as for the LAST ACT trial.

## Supplementary material

Supplementary File 1. Modified MRC grade of TBM.

Supplementary File 2. IRIS diagnostic criteria.

Supplementary File 3. Current standard of care in DILI.

Supplementary File 4. TBM diagnostic criteria.

Supplementary File 5. SPIRIT checklist.

Supplementary File 6. ACT HIV consent form for HTD Vietnam.
