# Supplementary material for: Adjunctive dexamethasone for the treatment of HIV-infected adults with tuberculous meningitis (ACT HIV): Study protocol for a randomised controlled trial
Source: Wellcome Open Res. 2018 Jun 20;3:31. Originally published 2018 Mar 20. [Version 2] doi: 10.12688/wellcomeopenres.14006.2 (PMC6143919; doi:10.12688/wellcomeopenres.14006.2)
Supplement: Supplementary file 2 [file wellcomeopenres-3-15979-s0001.tgz › a14e2851-9ab8-4248-a51a-67912b5ee6cc.docx]

**Immune reconstitution inflammatory syndrome (IRIS)**

**Case definition for paradoxical tuberculosis-associated IRIS [14]**

There are three components to this case definition:

**(A) Antecedent requirements**

Both of the two following requirements must be met:

- Diagnosis of tuberculosis: the tuberculosis diagnosis was made before starting ART and this should fulfil WHO criteria for diagnosis of smear-positive pulmonary tuberculosis, smear-negative pulmonary tuberculosis, or extrapulmonary tuberculosis
- Initial response to tuberculosis treatment: the participant’s condition should have stabilised or improved on appropriate tuberculosis treatment before ART initiation—eg, cessation of night sweats, fevers, cough, weight loss. (Note: this does not apply to participants starting ART within 2 weeks of starting tuberculosis treatment since insufficient time may have elapsed for a clinical response to be reported)

**(B) Clinical criteria**

The onset of tuberculosis-associated IRIS manifestations should be within 3 months of ART initiation, reinitiation, or regimen change because of treatment failure.

Of the following, at least one major criterion or two minor clinical criteria are required:

*Major criteria*

- New or enlarging lymph nodes, cold abscesses, or other focal tissue involvement—eg, tuberculous arthritis
- New or worsening radiological features of tuberculosis (found by chest radiography, abdominal ultrasonography, CT, or MRI)
- New or worsening CNS tuberculosis (meningitis or focal neurological deficit—eg, caused by tuberculoma)
- New or worsening serositis (pleural effusion, ascites, or pericardial effusion)

*Minor criteria*

- New or worsening constitutional symptoms such as fever, night sweats, or weight loss
- New or worsening respiratory symptoms such as cough, dyspnoea, or stridor
- New or worsening abdominal pain accompanied by peritonitis, hepatomegaly, splenomegaly, or abdominal adenopathy

**(C) Alternative explanations for clinical deterioration must be excluded if possible**

- Failure of tuberculosis treatment because of tuberculosis drug resistance
- Poor adherence to tuberculosis treatment
- Another opportunistic infection or neoplasm (it is particularly important to exclude an alternative diagnosis in participants with smear-negative pulmonary tuberculosis and extrapulmonary tuberculosis where the initial tuberculosis diagnosis has not been microbiologically confirmed)
- Drug toxicity or reaction
