# Supplementary material for: Adjunctive dexamethasone for the treatment of HIV-infected adults with tuberculous meningitis (ACT HIV): Study protocol for a randomised controlled trial
Source: Wellcome Open Res. 2018 Jun 20;3:31. Originally published 2018 Mar 20. [Version 2] doi: 10.12688/wellcomeopenres.14006.2 (PMC6143919; doi:10.12688/wellcomeopenres.14006.2)
Supplement: Supplementary file 3 [file wellcomeopenres-3-15979-s0002.tgz › d0b33f2e-a4ae-4777-af86-6c49cf76e692.docx]

**Current standard of care in drug-induced liver injury**

Current standard of care (current USA CDC guidelines [4]):

Stop RHZ immediately and add levofloxacin (moxifloxacin is acceptable alternative) and aminoglycoside (according to local practice and known resistance) to ethambutol. Restart R (at full dose) once transaminases are <2X ULN and no hepatitis symptoms. If no increase in transaminases after 7 days add isoniazid (at full dose) and stop levofloxacin and aminoglycoside. If transaminases remain normal on full dose R and H, Z was the likely cause and it should not be re-started and treatment duration should be extended to ≥12 months.

If transaminases rise ≥ 5x ULN, or ≥3x ULN with symptoms, at any time after re-introduction of R and/or H the physician should stop R and/or H (depending on which was associated with the transaminase rise). If neither R or H can be used, treat with levofloxacin, an aminoglycoside and ethambutol. If R can be used, but not H, treat with R, levofloxacin and ethambutol. If H can be used, but not R, treat with H, levofloxacin and ethambutol.

R = rifampicin

H = Isoniazid

Z = pyrazinamide
