# Supplementary material for: Adjunctive dexamethasone for the treatment of HIV-infected adults with tuberculous meningitis (ACT HIV): Study protocol for a randomised controlled trial
Source: Wellcome Open Res. 2018 Jun 20;3:31. Originally published 2018 Mar 20. [Version 2] doi: 10.12688/wellcomeopenres.14006.2 (PMC6143919; doi:10.12688/wellcomeopenres.14006.2)
Supplement: Supplementary file 4 [file wellcomeopenres-3-15979-s0003.tgz › 2148b794-72ca-4882-ad26-23908e51e0e6.docx]

**Diagnostic criteria for TBM**^23^

|  |  | | Diagnostic score |
| --- | --- | --- | --- |
| **Clinical criteria** | (Maximum category score=6) | | |
|  | Symptom duration of >5 days | | 4 |
|  | Systemic symptoms suggestive of tuberculosis (one or more of the following): weight loss (or poor weight gain in children), night sweats, or persistent cough for >2 weeks | | 2 |
|  | History of recent (within past year) close contact with an individual with pulmonary tuberculosis or a positive TST or IGRA (only in children <10 years of age) | | 2 |
|  | Focal neurological deficit (excluding cranial nerve palsies) | | 1 |
|  | Cranial nerve palsy | | 1 |
|  | Altered consciousness | | 1 |
| **CSF criteria** | (Maximum category score=4) | | |
|  | Clear appearance | | 1 |
|  | Cells: 10-500 per μl | | 1 |
|  | Lymphocytic predominance (>50%) | | 1 |
|  | Protein concentration >1 g/L | | 1 |
|  | CSF to plasma glucose ratio of less than 50% or an absolute CSF glucose concentration less than 2.2mmol/L | | 1 |
| **Cerebral imaging criteria** | (Maximum category score=6) | | |
|  | Hydrocephalus | | 1 |
|  | Basal meningeal enhancement | | 2 |
|  | Tuberculoma | | 2 |
|  | Infarct | | 1 |
|  | Pre-contrast basal hyperdensity | | 2 |
| **Evidence of tuberculosis elsewhere** | (Maximum category score=4) | | |
|  | Chest radiograph suggestive of active tuberculosis: signs of tuberculosis=2; miliary tuberculosis=4 | | 2 or 4 |
|  | CT/ MRI/ ultrasound evidence for tuberculosis outside the CNS | | 2 |
|  | AFB identified or *Mycobacterium tuberculosis* cultured from another source-i.e., sputum, lymph node, gastric washing, urine, blood culture | | 4 |
|  | Positive commercial *M. tuberculosis* NAAT from extra-neural specimen | | 4 |
| **Diagnostic criteria based on total score:**  Possible TBM: score 6-9 (if no brain imaging) or 6-11 (if brain imaging)  Probable TBM: score >9 (if no brain imaging) or >11 (if brain imaging)  Definite TBM: acid-fast bacilli seen in CSF or *M. tuberculosis* cultured or detected by commercial NAAT in CSF | |  |  |
