# Supplementary material for: Adjunctive dexamethasone for the treatment of HIV-infected adults with tuberculous meningitis (ACT HIV): Study protocol for a randomised controlled trial
Source: Wellcome Open Res. 2018 Jun 20;3:31. Originally published 2018 Mar 20. [Version 2] doi: 10.12688/wellcomeopenres.14006.2 (PMC6143919; doi:10.12688/wellcomeopenres.14006.2)
Supplement: Supplementary file 6 [file wellcomeopenres-3-15979-s0005.tgz › 88484efd-ab5b-4054-b1dc-5261ee6f896c.docx]

**PATIENT INFORMATION SHEET: A TRIAL OF STEROIDS FOR TB MENINGITIS**

**For patients enrolled in HTD, Vietnam only**

**Adjunctive Corticosteroids for Tuberculous meningitis in HIV-infected adults (The ACT HIV trial)**

You are being invited to take part in a research study of adults with tuberculous (TB) meningitis. Approximately 520 people will be a part of this study which is being conducted by the Oxford University Clinical Research Unit (OUCRU) in Vietnam. The study is being performed in two hospitals in Ho Chi Minh City, Vietnam, and two hospitals in Jakarta, Indonesia.

Please read this information sheet carefully or have someone read it to you. You will be given a copy of this form to keep.

**What is the reason for doing the study?**

TB-meningitis is an infection of the brain caused by tuberculosis. It is a severe disease. We want to improve the treatment of TB-meningitis. Normally, TB-meningitis patients will be given antibiotics for at least 9 months to kill the infection. However, we want to know whether we should add a drug – called dexamethasone – to help control the inflammation in the brain at the start of treatment whilst the antibiotics kill the infection. It is possible that if we control the inflammation with dexamethasone more patients will recover from TB meningitis, but we don’t know whether it works. That is why we are doing this study.

There is another reason for doing the study. One of the problems with the standard antibiotics for tuberculosis treatment is that they can upset your liver. In about one person in ten, the liver can become inflamed by the drugs and the drugs may need to be stopped. If the drugs are stopped the infection in the brain can come back. We want to work out what to do if your liver becomes inflamed. We want to see which of three different options for dealing with the liver inflammation is best.

Lastly, we want to work out how dexamethasone might work to help patients with TB patients. Therefore, with your consent, we would like to monitor how you get better very carefully. We will take X-ray images of your brain and we will use ultrasound to measure the pressure in your brain and the amount of fluid in your body. We also want to see what effect dexamethasone has on the hormones in your body. None of these interventions will cause you pain or discomfort, but they will allow us to monitor you very closely and to detect and treat any complications of the disease should they occur.

**What will happen if I take part in the study?**

There are two groups of patients in this study. You have an equal chance to be assigned to one of these two groups (it is *random*). One will be given standard antibiotic treatment for TB meningitis with dexamethasone for the first 6-8 weeks of treatment. The other group with be given standard antibiotic treatment for TB meningitis without dexamethasone. However, patients in this second group will receive injections and pills that will look identical to the dexamethasone injections and pills given to the first group, but they will not contain any drug (they are harmless ‘placebos’). Neither you nor the treating hospital staff will know which group you are in and whether you receive dexamethasone or placebo. At the end of the trial the investigators will find out which treatment you and everyone else in the trial received and they will be able to calculate whether dexamethasone helped patients get better, or not. This is called “a double blind, randomized placebo controlled trial” and is the best way to study a new treatment, because it gives the most accurate answer on which treatment is best.

All patients treated for TB meningitis will stay in hospital for the period of treatment until their health status remains stable and they are able to be discharged. While you are in hospital, you will be closely monitored by the study doctors. We want to understand how dexamethasone might work; therefore we will follow your response to treatment very closely. As long as it is safe to do so, we will scan your brain at the start of the study and after 2 and 9 months. These scans will help us monitor your treatment carefully and allow us to detect and manage any complications quickly. They do not cause any pain or discomfort. If you would like to participate in the trial but do not agree to having brain images taken, you can decline consent by checking the checkbox on the consent form.

In addition, we will perform tests on your blood and urine to monitor the amount of salt in your body in the first few weeks of your treatment in hospital and to see how dexamethasone affects the hormones in your body. Patients with TB meningitis often lose salt and this can make them very unwell. We want to work out why this happens and what to do about it. We will use an ultrasound machine (which is harmless and does not cause any pain or discomfort) to see how much fluid you have in your body. We will also use the ultrasound machine to see if your brain pressure is high by looking at the back of your eyes. In addition, we will test how dexamethasone affects the hormones produced by your adrenal gland (a small gland just above the kidney which produces steroid hormones). We will do this by giving you a small injection of a substance that stimulates the adrenal gland to produce hormones. We will test your blood before and after the injection and see whether dexamethasone has changed the way the adrenal gland produces hormones. The test is completely safe and will be performed after 3 weeks and at the end of dexamethasone treatment.

If you would like to participate in the trial but do not agree to having the salt studies and ultrasounds performed, or the hormone study, you can decline consent by checking the checkbox on the consent form.

Regular care for TB meningitis includes blood tests and tests on the spinal fluid in your back. To take spinal fluid, doctors will put a needle in your back. We do these tests to make sure we are treating you with the right antibiotics and to assess whether you are getting better. Blood and spinal fluid samples taken for study purposes will be taken as the same time as routine tests. Therefore, you don’t get extra needle pokes for these tests. The total amount of blood taken for research tests is about 57.5mls. We will also check a stool sample to make sure that you do not have a common parasite infection that may complicate your TB meningitis treatment. If you have this infection, we will treat it in the normal way.

After you leave hospital, you will be given TB medicine to take every day and followed up in the clinic every month. You may also be given study tablets to take for a few days or weeks. It is very important you take TB and study medicines as instructed. During your visits, some tests will be done to check your health.

The entire study lasts 24 months when treatment and testing will be finished. Your treatment for TB meningitis will last 9-12 months, regardless of whether you enter the study.

If you need treatment for HIV or any other condition during the trial, this will be given in the normal way. Being in the trial will not affect how you are treated for any other health problem you might have.

If your liver gets upset by the tuberculosis antibiotics at any time during treatment we may need to stop the drugs. We are not sure of the best way to stop the drugs: whether to stop all of them immediately, or wait and just stop one or two. Therefore, with your consent, if your liver does get upset one of these three different drug-stopping options will be selected for you. Like the dexamethasone treatment, which option you receive will be by chance (random). We believe all three options are safe, but some of the options may allow you to have the best antibiotics for longer whilst still allowing your liver to recover. If you would like to participate in the trial but do not agree to taking part in this part of the trial, you can decline consent by checking the checkbox on the consent form.

**What tests will be done and what are their risks?**

Having a blood test taken can be uncomfortable and may cause a bruise. Having spinal fluid taken may make your back hurt. However these procedures are essential to diagnose and follow the disease whether or not you enter the study. The doctors will ask for your consent on each occasion that spinal fluid is taken. There is no extra spinal fluid is taken as part of this study. The amount of extra blood taken for research tests should not affect your health. Your doctor will monitor your blood levels carefully and will not do extra tests if they think it will harm you.

All samples taken will be labeled with a study number rather than your name, to protect your identity. Samples will be tested or stored indefinitely in a freezer. Some tests that cannot be done in Viet Nam or Indonesia may be sent to laboratories outside of the country. If you agree, further tests on stored samples may be done in the future to improve our understanding of TB meningitis. These tests may include genetic testing. We want to see whether your genes influence the way you get better from the infection. If you would like to participate in the study but do not agree to have your samples stored for later genetic testing, you can decline consent to these tests by checking the checkbox on the consent form.

We will use image your brain with MRI when possible. This does not have any harmful side-effects and is painless. Likewise, the ultrasound tests that we will perform (to assess body fluid and brain pressure) are harmless and painless. The salt tests on blood and urine will be done at the same time as your regular standard blood tests. The hormone tests will require two small injections and three small samples of blood to be taken before and after each injection. The tests are safe with no side effects other than the discomfort of the needles. We also want to do some genetic tests on genes involved in immune responses and treatment outcomes to see whether your genes influence the way you get better from the infection.

**What are the drug side effects?**

All medicines can have side-effects, including the standard antibiotics given for tuberculosis treatment. Common side effects of the tuberculosis drugs include red coloration of urine (which is completely harmless and goes away when the drugs stop) and liver inflammation which can cause nausea, vomiting, and abdominal pain.

Doctors give dexamethasone to many patients for many different medical reasons and the side-effects are well known. However, when used for short periods (a few weeks, as in this trial) the side-effects of dexamethasone are generally uncommon and mild. Some people experience agitation or confusion with difficulty sleeping, blood sugars can become elevated, and dexamethasone can increase the risk of bleeding from the stomach. Sometimes, dexamethasone can cause people to gain weight and their blood pressure can increase. However, previous trials of dexamethasone in patients with TB meningitis have reported that side-effects are rare and it is a very safe treatment. However, if you do suffer from a side-effect that may be related to dexamethasone, the study medication will be stopped immediately and all necessary treatment will be given to you.

**Participation**

Being in a research study is your decision. If you do not want to be in the study, or at any time in the study decide to stop being in the study, the doctors will respect your decision. Your medical treatment will not be affected by your decision.

If you agree to be in the study you will be responsible for following the study schedule of tests and taking your drugs. You will also be responsible for coming to the monthly visits until completing 9-12 months of treatment and the occasional follow-up visits occurring after that for another 12 months.

**Benefits and Costs**

This study may not have a direct benefit to you as it is not yet known if the study treatment will improve the treatment of patients with TB meningitis. The results from this study will help determine the best way to treat patients with this disease in the future.

You will not have to pay for any extra costs if you participate in the study. The drugs and tests which are a part of this study will be paid for by the study sponsor. If you decide to be in the study, the cost of your hospital care for tuberculosis meningitis will be covered by the study until you finish or stop participation in the study.

When you come to the hospital for follow-up visits you will be supported for your travel costs. The doctor will explain to you the compensation you will receive for travel.

**Confidentiality**

All information about you will be kept confidential. Your medical records will be reviewed in strict confidence by those who are working on this study and may also be reviewed by the ethics committees and health authorities reviewing the study. Your name will not be used on any of the study documents or on the stored blood samples or in any reports or publications about this study.

**Questions**

You are encouraged to ask any questions related to this study during the time of participation. If you have any other questions about the study, its procedures, risks and benefits, or alternatives please contact: {Dr. Name} at {Dr. Number}.

If you have any questions about your rights as a subject in this study, you may want to talk to {Dr. Name}, or if you want to speak to someone outside of the program you may contact the Ethics Committee at the Hospital for Tropical Diseases at 08 39238704.

If you have general questions, please call the OUCRU Clinical Research Unit at +84 3924 1983.

Thank you for your time and your consideration to participate in this study.

**INFORMED CONSENT FORM**

**For patients enrolled in HTD only**

**Adjunctive Corticosteroids for Tuberculous meningitis in HIV-infected adults (The ACT HIV trial)**

I have read the information given to me and freely agree to be in this study. I also have had a chance to discuss it with the study staff.

I have been told about the risks and benefits. I got answers that I could understand to all my questions.

I consent to study staff collecting information about my health and using this information for future medical research.

I agree that testing on some samples may be done outside of Viet Nam or Indonesia.

I agree that all information collected on me during the trial can be made available to others in the future (open access) provided no one can identify me from the details provided.

I understand that I can withdraw from the study at any time. If I stop the study, it will **not** affect my future care. If I decide to stop the study, I agree that the information collected up to the point when I stop, may continue to be used.

**□ Yes or □ No** I agree that the samples taken can be stored for future testing.

**□ Yes or □ No** I agree that the samples taken can be used for genetic testing.

**□ Yes or □ No** I agree that if my liver gets upset by the anti-tuberculosis drugs I will participate in the study of how best to manage this complication.

**□ Yes or □ No** I agree to be in the brain imaging study.

**□ Yes or □ No** I agree to studies of salt in my body, including the use of ultrasound to asses my body fluid and brain pressure.

**□ Yes or □ No** I agree to be in the hormone study.

Screening Number [__][__][__][__][__]

By signing my name here, I confirm what is written above and that I have a copy of this form to keep until my part in the study ends.

| x_______________________ | x___________________ | ___/____/_____ |
| --- | --- | --- |
| Participant Signature | Print Name | Date of Signature |

**OR** – If someone else gives consent on behalf of the participant:

| Patient’s name: | x ___________________________________________ | | |
| --- | --- | --- | --- |
| Signature of Person Giving Consent:  x_______________ | Print Name:  x_____________ | Relationship to Participant:  x______________ | Date of Signature:  ____/____/_____ |

I, the undersigned, have fully explained the relevant information of this study to the person named above and will provide her/him with a copy of this signed and dated informed consent form.

| x___________________ | x_______________ | ___/____/_____ |
| --- | --- | --- |
| Investigator/Designee Signature | Print Name | Date of Signature |

**If the person giving consent cannot read the form themselves, a witness who is independent of the study must be present and sign here:**

I was present throughout the entire informed consent process with the participant. This form was read accurately to the volunteer, all questions from the volunteer were answered and the volunteer has agreed to take part in the research.

| x_______________ | x_____________________ | _____/______/_____ |
| --- | --- | --- |
| Witness Signature | Print Name | Date of Signature |
