# Supplementary material for: Adjunctive dexamethasone for the treatment of HIV-infected adults with tuberculous meningitis (ACT HIV): Study protocol for a randomised controlled trial
Source: Wellcome Open Res. 2018 Jun 20;3:31. Originally published 2018 Mar 20. [Version 2] doi: 10.12688/wellcomeopenres.14006.2 (PMC6143919; doi:10.12688/wellcomeopenres.14006.2)
Supplement: Peer review history

# Peer review: Referee response for version 2

**DOI:** 10.21956/wellcomeopenres.15979.r33814

**Published:** 2018-09-17

**Authors:** Ira Shah, Srushti Gandhi

**Affiliations:** 1 Medical Officer, Pediatric TB Clinic, B J Wadia Hospital for Children, Mumbai, Maharashtra, India; 2 Department of Paediatrics, HOD Pediatric Infectious Diseases, Incharge, Pediatric HIV and TB clinic, Bai Jerbai Wadia Hospital for Children, Mumbai, Maharashtra, India

A study on adjunctive dexamethasone for the treatment of HIV-infected adults with tuberculous meningitis (ACT HIV) is much needed and could prove to be of immense benefit.

- *T* *he exclusion criteria includes:*

*“b) More than 6 consecutive days of two or more drugs active against immediately before screening.*

*c) More than 3 consecutive days of any type of orally or intravenously administered corticosteroid immediately before randomisation”*

 1. It would help to mention an interval of how many hours-days is required to be eligible for the study in case the person has received any of the afore-mentioned drugs prior to the study.

 2. Does the inclusion criteria include persons with TBM as a part of disseminated TB? 

  

- *The Ancillary study 6:* *Pathophysiology and treatment of hyponatraemia and raised intracranial pressure*
- does not specify what modalities will be used to determine the best method of treatment.

 

 4. Will the ART be modified if the patient develops DILI?

 5. Interactions with Rifampicin and EFV are also seen. Are you going to monitor the TDM of efavirenz when using rifampicin in these patients?

 6. What about co-infection with HBV and HCV? Are you going to include these patients? It is not mentioned in the inclusion or exclusion criteria.

 7. Other co-infections in HIV infected (which are non-neurological) is also not mentioned. Some patients do have systemic fungal infections such as esophageal candidiasis and histoplasmosis. Giving dexamethasone in these patients will be a problem. Are you going to include these patients as well as?

 8. Survival in HIV infected patients with TBM will also depend on baseline CD4 count and HIV viral load. So that may be a confounding factor when analysing the data. This issue needs to be addressed.

 9. In case of DILI in the intensive phase, a regime of Lfx, E and aminoglycoside may not be enough. Would you consider addition of Linezolid or any other drug that is not hepatotoxic and does not have interaction with the ART.

 Overall, the study protocol is well written.

We have read this submission. We believe that we have an appropriate level of expertise to confirm that it is of an acceptable scientific standard, however we have significant reservations, as outlined above.

# Peer review: Referee response for version 2

**DOI:** 10.21956/wellcomeopenres.15979.r33350

**Published:** 2018-07-17

**Authors:** Rohit Bhatia, Manish Modi

**Affiliations:** 1 Department of Neurology, All India Institute of Medical Sciences (AIIMS), New Delhi, Delhi, India; 2 Postgraduate Institution of Medical Education and Research, Chandigarh, India

We thank authors for their reply to our concerns

 However, we still have some concerns regarding the protocol;

 1. Q 1. We still have significant concerns about the DILI substudy arm1 for safety issues.

 2. Q.4. We disagree with the authors that imaging is not being done as a standard for all patients. As a part of the study, at least a CT scan and MRI where necessary among all patients should be mandatory in view that all of them are HIV positive patients and could have infection other than TB as well as to detect complications.

 3. Q7. Since the inclusion criteria is patients with HIV, we believe that most patients would hopefully be on treatment already at the time of there presentation with suspected TBM. We understand that ART is typically advised  after an interval of starting ATT among patients with and TBM newly diagnosed HIV or known HIV without any ART therapy,  in view of the previous trial results as quoted by the author. However steroid was not the intervention in that trial. Our concern whether an earlier ART is to be considered was because one group of patients are being given dexamethasone, which may have a concern of worsening the underlying HIV disease. The best evidence from the previous trial and recommendations of experts should be taken to decide about the best time to start ART  in this trial, as we understand that this a RCT and one group is free from dexamethasone which of course may complicate the study design. However, further expert opinion is warranted.

We have read this submission. We believe that we have an appropriate level of expertise to confirm that it is of an acceptable scientific standard, however we have significant reservations, as outlined above.

# Response

**Published:** 2018-07-25

**Authors:** Joseph Donovan

**Affiliations:** Oxford University Clinical Research Unit, Ho Chi Min City, Vietnam

Question 1

We feel we have already addressed this question and no further details are given as to why there are safety concerns regarding arm 1 of the DILI sub-study. There is no evidence to suggest that DILI arm 1 is unsafe, and the purpose of clinical research such as this is to produce high quality evidence to support our clinical treatment. We do not know that it is safer to stop essential first line anti-tuberculous drugs at an ALT threshold of >5x ULN, and change to less effective anti-tuberculous therapy. This is frequently done but we do not know it is the safer approach in TBM. As previously explained, it is anticipated that arm 1 (continue all 4 first line anti-TB drugs) and arm 2 (stop only pyrazinamide) of the DILI study will result in less adverse events due to the continuation of the best and most appropriate anti-TB drugs. There are clear safety thresholds where a patient in DILI arms 1 or 2 moves to arm 3. Patients are reviewed daily and LFTs, including INR, are checked regularly. This trial has received ethical approval from the Oxford Tropical Research Ethics Committee, the Ethics Committees of the Hospital for Tropical Diseases and Pham Ngoc Thach Hospital, the Vietnam Ministry of Health, and Faculty of Medicine University of Indonesia. An independent data monitoring committee will review unblinded trial data at interim analysis points and advise on any safety issues. We believe the DILI sub-study is important, and there is no evidence base to say that arm 1 is unsafe.

Question 2

CT or MRI brain imaging can be important in the management of a patient with TBM, but it is not always available. It may be ideal to have imaging for every patient we treat but this is not always possible in all settings. Clinical trials cannot only be performed in sites where gold standard level testing is available for every patient. Where availability of imaging is limited, imaging must be done on a case-by-case basis where it is required.

Question 3

The experience of the reviewers suggests that most patients would be on anti-retroviral treatment at the time of presentation with TBM, however this is not the case in our setting. We do not believe that corticosteroid therapy will worsen HIV infection, and corticosteroids are often given to HIV co-infected individuals for a multitude of reasons. Furthermore, this is a double blind trial and physicians will not know who is treated with dexamethasone. The reviewers say that “the best evidence from the previous trial and recommendations of experts should be taken to decide about the best time to start ART in this trial”. Our protocol states that ART in both Ho Chi Minh City and Jakarta is often commenced 6–8 weeks after the start of anti-tuberculosis treatment, “with the precise timing left to the discretion of the attending physician”. Our trial physicians also follow local expert guidelines. We do not believe that in the absence of additional clinical trial data further expert guidance is needed.

Yours Sincerely

Professor Guy Thwaites (Senior author and study Chief Investigator)

Dr Joseph Donovan (First author and Research Fellow)

# Peer review: Referee response for version 1

**DOI:** 10.21956/wellcomeopenres.15222.r33086

**Published:** 2018-06-08

**Authors:** Rohit Bhatia, Manish Modi

**Affiliations:** 1 Department of Neurology, All India Institute of Medical Sciences (AIIMS), New Delhi, Delhi, India; 2 Postgraduate Institution of Medical Education and Research, Chandigarh, India

The protocol is well written. Our concerns and queries are outlined below.

1. DILI study:  A concern to the reviewers is the co-study of DILI related interventions within the same trial. Since these patients are HIV infected, with adjuvant therapies and co-morbidities, further randomization to the groups may increase the likelihood of adverse events and outcomes especially with the allocation strategies planned.
2. Is the DILI study strategy 1 safe.  Could the authors consider strategy 2 and 3 only.
3. Many ancillary studies are planned from the same data. Will the patients be analyzed within the same groups as allocated based on the dexamethasone intervention allocation. The same is not clear.
4. The authors have planned to do imaging at intervals. The authors may kindly clarify whether imaging is being done at baseline for all patients for exclusion of diagnosis other than TBM and also to ascertain presence of features suggestive of TBM and features like hydrocephalus, tuberculomas, infarcts etc., as this is extremely important. An imaging at the end of the study may be desirable to ascertain resolution of the disease.
5. The authors have planned on an ATT regimen excluding streptomycin. What about patients with basal visual complications. Is there a provision to alter the regimen by replacing streptomycin instead of ethambutol in such group of patients?
6. The authors plan to use RHE regimen in the maintenance phase. Would authors consider to use pyrazinamide instead of ethambutol in the maintenance phase, considering the higher CNS bioavailability of this drug.
7. The authors plan to start ART after 4-6 weeks of the diagnosis. Considering that steroids are being used in this trial, would the authors consider an earlier ART intervention than planned? Is there a shorter period that is safe? What about a patient who is already a diagnosed case of HIV on ART. Will he/she be excluded from the study.
8. Is Pregnancy an exclusion criteria.
9. It is reasonable to exclude presence of non-tuberculous mycobacteria and also presence of MDR TB at baseline by best available modalities to ascertain diagnostic certainty and therapeutic responsiveness.
10. Will participants with geneXpert suggestive of rifampicin resistance be modified in the ATT regimen or will be excluded from the study?
11. Other complications like opto-chiasmatic arachnoiditis and myelitis should also be included in the outcomes as dexamethasone use is reported to help in these complications.

 

We have read this submission. We believe that we have an appropriate level of expertise to confirm that it is of an acceptable scientific standard, however we have significant reservations, as outlined above.

# Response

**Published:** 2018-06-15

**Authors:** Joseph Donovan

**Affiliations:** Oxford University Clinical Research Unit, Ho Chi Min City, Vietnam

Dear Dr Bhatia and Dr Modi,

Thank you very much reviewing our ACT HIV trial protocol. I would like to address each of your points individually.

**Question 1**

DILI study:  A concern to the reviewers is the co-study of DILI related interventions within the same trial. Since these patients are HIV infected, with adjuvant therapies and co-morbidities, further randomization to the groups may increase the likelihood of adverse events and outcomes especially with the allocation strategies planned.

Author reply

DILI related interventions are conducted within the same trial, but the DILI strategies (specifically arms 1 and 2, compared with the routine care arm 3) are not expected to increase adverse events. It is in fact anticipated that arm 1 (continue all 4 first line anti-TB drugs) and arm 2 (stop only pyrazinamide) of the DILI study will result in less adverse events due to the continuation of the best and most appropriate anti-TB drugs. With regard to liver function, this will be closely monitored. We accept that patients in the DILI study may be on concurrent ART. However, DILI occurs more frequently in individuals with HIV therefore it is very important that DILI interventions are assessed in an HIV positive group, to avoid unnecessary cessation of first line anti-TB drugs if arms 1 and / or 2 are effective. From a safety perspective, if a patient develops DILI, prior randomisation to dexamethasone or placebo should not affect DILI in an adverse way.

If DILI develops ART should not be stopped until all other management has been tried (including interrupting anti-tuberculosis drugs according to the randomised schedule). HIV specialists and liver specialists should be consulted at this point. Patients will receive regular clinical and blood test monitoring, and an independent data monitoring committee will review unblinded trial data at interim analysis points.  Adverse events due to DILI will be recorded for the DILI study but also for the primary intervention (dexamethasone or placebo). An increased number of adverse events in DILI arms 1 and 2 are not expected, but if they do occur it will be made clear in the analysis if they were associated with DILI randomisation, although these adverse events will also be reported for the primary intervention arm in which they occurred. 

**Question 2**

Is the DILI study strategy 1 safe?  Could the authors consider strategy 2 and 3 only?

Author reply

The authors believe that DILI strategy 1 is safe, and this sub study has received ethical approval. Patients will undergo regular clinical and blood test monitoring, and there is a clear procedure to follow for moving patients from strategy 1 to strategy 3. An independent data monitoring committee will review unblinded data from this trial at interim analysis time points and advise the trial steering committee on any safety issues. The authors are not aware of any evidence suggesting a lack of safety of arm 1 of the DILI study compared with arm 3. Stopping rifampicin isoniazid and pyrazinamide (strategy 3) may in fact be the least safe option in TBM due to discontinuation of the most important drugs for the treatment of brain infection.

**Question 3**

Many ancillary studies are planned from the same data. Will the patients be analyzed within the same groups as allocated based on the dexamethasone intervention allocation. The same is not clear.

Author reply

Ancillary studies will be analysed within the same groups as allocated based on the dexamethasone intervention allocation. Allocation to the intervention (dexamethasone or placebo) is important for each sub study, given the effect of corticosteroids on inflammatory pathways, diabetes control, Strongyloides infection, raised intracranial pressure and adrenal suppression. For the DILI study patients will be analysed in their DILI arm, but an additional adjusted analysis (with adjustment for the initial randomisation) will also be performed.

**Question 4**

The authors have planned to do imaging at intervals. The authors may kindly clarify whether imaging is being done at baseline for all patients for exclusion of diagnosis other than TBM and also to ascertain presence of features suggestive of TBM and features like hydrocephalus, tuberculomas, infarcts etc., as this is extremely important. An imaging at the end of the study may be desirable to ascertain resolution of the disease.

Author reply

All patients recruited to this study at HTD in Vietnam will have MRI brain imaging at day 0, day 60 and at 1 year (assuming they consent to the MRI ancillary study). At the other sites, brain imaging is performed based upon the routine care offered for brain infection at that site. In many cases brain imaging will be performed in order to establish the diagnosis and investigate for complications. However it is accepted that brain imaging is not always readily available at all study sites, and this study does not mandate brain imaging for every patient. In the context of an unclear clinical presentation, or clinical signs where brain imaging is required, the study team will strongly support the decision of the treating physician to conduct brain imaging.

**Question 5**

The authors have planned on an ATT regimen excluding streptomycin. What about patients with basal visual complications. Is there a provision to alter the regimen by replacing streptomycin instead of ethambutol in such group of patients?

Author reply

First line anti-tuberculosis treatment will follow current Vietnamese and Indonesian national guidelines, where rifampicin, isoniazid, pyrazinamide and ethambutol will be given for at least the first 2 months of treatment where there is no drug resistance. Patients with visual complications will discontinue ethambutol and an alternative drug will be used in its place. The decision of this 4 ^th^ drug will be made by the treating physician, and is not mandated by the trial. In practice it will be either a fluoroquinolone or streptomycin.

**Question 6**

The authors plan to use RHE regimen in the maintenance phase. Would authors consider to use pyrazinamide instead of ethambutol in the maintenance phase, considering the higher CNS bioavailability of this drug?

Author reply

First line anti-tuberculosis treatment will follow current Vietnamese and Indonesian national guidelines. Rifampicin, isoniazid, pyrazinamide and ethambutol will be given for at least the first 2 months of treatment where there is no drug resistance. Pyrazinamide will then be stopped and rifampicin, isoniazid and ethambutol will be given until month 12. This trial does not seek to assess alternative anti-tuberculous drug treatment strategies and anti-tuberculous therapy will follow national guidelines. 

**Question 7**

The authors plan to start ART after 4-6 weeks of the diagnosis. Considering that steroids are being used in this trial, would the authors consider an earlier ART intervention than planned? Is there a shorter period that is safe? What about a patient who is already a diagnosed case of HIV on ART. Will he/she be excluded from the study?

Author reply

Immediate commencement of ART (compared with ART commenced after 2 months) in TBM has been associated with increased grade 4 adverse events, supporting deferred commencement of ART (Torok et al. Clin Infect Dis. 2011 Jun; 52(11): 1374–1383). The optimal time to start ART in TBM is not known, depends upon TBM disease severity and CD4 count, and varies between centres regarding routine practice. Exactly when to start ART is decided upon by the treating physician, although this is often between 6 and 8 weeks after TBM diagnosis. There is no evidence to guide whether a shorter period (for example 2 weeks) is safe in TBM. Only 50% of patients in this trial receive corticosteroids, and as the trial is double blinded these patients cannot be identified. Therefore adjunctive corticosteroid use cannot be used to support starting ART earlier. A patient with known HIV already on ART will continue this ART when they enter the trial, and during the trial.

**Question 8**

Is Pregnancy an exclusion criteria?

Author reply

No, pregnancy is not an exclusion criteria.

**Question 9**

It is reasonable to exclude presence of non-tuberculous mycobacteria and also presence of MDR TB at baseline by best available modalities to ascertain diagnostic certainty and therapeutic responsiveness.

Author reply

Exclusion of non-tuberculous mycobacteria infection and MDR TBM would be an alternative way to run this trial. However we would like to enroll those adult HIV-infected patients who have a clinical diagnosis of TBM and assess the effect of corticosteroids. MDR TBM patients are an important part of this group and will be included. If infection with non-tuberculous mycobacteria is suspected then the patient will not be enrolled (this would be an alternative brain infection, and this is an exclusion criteria). If non-tuberculous mycobacteria are later identified as the cause of disease (instead of TBM) the patient will still form part of the intention to treat analysis in the arm in which they were randomised.

**Question 10**

Will participants with geneXpert suggestive of rifampicin resistance be modified in the ATT regimen or will be excluded from the study?

Author reply

Patients with a positive GeneXpert result consistent with rifampicin resistance will receive MDR TBM treatment as per national guidelines, in conjunction with a regional specialist. They will not be excluded from the main study. They will however not be eligible for the DILI study.

**Question 11**

Other complications like opto-chiasmatic arachnoiditis and myelitis should also be included in the outcomes as dexamethasone use is reported to help in these complications.

Author reply

If these conditions are detected they will be recorded. However they do not form part of the outcomes of this trial (unless they lead to focal neurological deficits or a decrease in GCS as per the criteria in the protocol).

# Peer review: Referee response for version 1

**DOI:** 10.21956/wellcomeopenres.15222.r32220

**Published:** 2018-03-26

**Authors:** Ravindra K Garg

**Affiliations:** 1 Department of Neurology, King George's Medical University, Lucknow, Uttar Pradesh, India

Adjunctive dexamethasone for the treatment of HIV-infected adults with tuberculous meningitis is a pertinent issue which still need to be answered. The protocol is well written and the study will be

 conducted by known experts of the field. How will authors deal with  complications of TBM, like hydrocephalus, stroke, tuberculomas  and vision loss at inclusion and follow up, is not very clear cut. A

 neuroimaging at inclusion I suggest should  be done in all cases, other wise many of these complications will be missed. Is there any provision for shunt surgery?

I have read this submission. I believe that I have an appropriate level of expertise to confirm that it is of an acceptable scientific standard.
